# Prokaryotic soluble overexpression and purification of oncostatin M using a fusion approach and genetically engineered *E. coli* strains

**DOI:** 10.1038/s41598-019-50110-6

**Published:** 2019-09-23

**Authors:** Minh Tan Nguyen, Musharrat Jahan Prima, Jung-A. Song, Julee Kim, Bich Hang Do, Jiwon Yoo, Sangsu Park, Jaepyeong Jang, Sunju Lee, Eunyoung Lee, Michelle de Paula Novais, Hyeon-Beom Seo, Seon-yeong Lee, Mi-La Cho, Chong Jai Kim, Yeon Jin Jang, Han Choe

**Affiliations:** 1 Department of Physiology, Asan-Minnesota Institute for Innovating Transplantation, Bio-Medical Institute of Technology, University of Ulsan College of Medicine, Asan Medical Center, Seoul, 05505 Korea; 2 NTT Hi-Tech Institute, Nguyen Tat Thanh University, Ho Chi Minh city, Vietnam; 3 Faculty of Pharmacy, Ton Duc Thang University, Ho Chi Minh city, Vietnam; 4 The Rheumatism Research Center, The Laboratory of Immune Network, CRCID, The Catholic University of Korea, Seoul, 06591 Korea; 5 Department of Pathology, Asan-Minnesota Institute for Innovating Transplantation, Asan Medical Center, University of Ulsan College of Medicine, Seoul, 05505 Korea

**Keywords:** Oncostatin M, Proteins

## Abstract

Human Oncostatin M (OSM), initially discovered as a tumour inhibitory factor secreted from U-937 cells, is a gp130 (IL-6/LIF) cytokine family member that exhibits pleiotropic effects in inflammation, haematopoiesis, skeletal tissue alteration, liver regeneration, cardiovascular and metabolic diseases. Cytoplasmic expression of OSM in *Escherichia coli* results in inclusion bodies, and complex solubilisation, refolding and purification is required to prepare bioactive protein. Herein, eight N-terminal fusion variants of OSM with hexahistidine (His6) tag and seven solubility-enhancing tags, including thioredoxin (Trx), small ubiquitin-related modifier (Sumo), glutathione S-transferase (GST), maltose-binding protein (MBP), N-utilisation substance protein A (Nusa), human protein disulphide isomerase (PDI) and the b‘a’ domain of PDI (PDIb‘a’), were tested for soluble OSM expression in *E. coli*. The His6-OSM plasmid was also introduced into genetically engineered Origami 2 and SHuffle strains to test expression of the protein. At 18 °C, MBP-tagged OSM was highly expressed and solubility was dramatically enhanced. In addition, His6-OSM was more highly expressed and soluble in Origami 2 and SHuffle strains than in BL21(DE3). MBP-OSM and His6-OSM were purified more than 95% with yields of 11.02 mg and 3.27 mg from a 500 mL culture. Protein identity was confirmed by mass spectroscopy, and bioactivity was demonstrated by *in vitro* inhibition of Th17 cell differentiation.

## Introduction

Human Oncostatin M (OSM) was initially discovered as a tumour inhibitory factor secreted by phorbol 12-myristate 13-acetate (PMA)-treated U-937 cells, a histiocytic lymphoma cell line^1^. In a follow-up study, sequence analysis of cDNA clones isolated from mRNA from U-937 cells revealed that OSM is a 252 amino acid glycoprotein with a hydrophobic signal peptide at its N-terminus^2^. Although the full-length protein is biologically active, proteolytic processing of a hydrophilic C-terminal peptide results in a mature form of OSM with higher biological activity^3,4^. OSM is grouped into the gp130 (IL-6/LIF) cytokine family based on shared structural and functional properties^5,6^. Among the cytokine family members, OSM and leukaemia inhibitory factor (LIF) are the most closely related (27% sequence similarity), and the genes encoding these proteins are located in close proximity on chromosome 22^7,8^.

Several studies have indicated that OSM is able to bind to two distinct functional receptor complexes: heterodimers of gp130 with either the LIF receptor or the unique OSM receptor beta^6,9^. The binding of OSM to either of these receptor complexes results in the stimulation of intracellular signal cascades, including the JAK/STAT3, MAPK and PI3K/AKT pathways^10–12^. Although OSM was initially described as a growth modulator of tumour cells^1^, the binding of OSM to its receptors exerts multifunctional actions upon a wide variety of cells in multiple organs and tissues^13^. Indeed, extensive research has shown that OSM exhibits pleiotropic effects in inflammation, haematopoiesis, skeletal tissue alteration, liver regeneration, cardiovascular and metabolic diseases^4,14^. However, despite a considerable body of research, the exact pathological and physiological functions of OSM are not yet fully understood due to its pleiotropic characteristics^4,15^.

OSM is primarily produced by activated T cells, monocytes, macrophages and dendritic cells^16^, but its levels in plasma are extremely low (24 pg/mL)^14^. Since it was first isolated from U-937, several attempts have been made to heterologously produce recombinant OSM in *E. coli*^17^, yeast^14^ and Chinese Hamster Ovary (CHO) cells^18^, with yields differing between host organisms. In general, due to its high yields and inexpensive culturing process, *E. coli* is the most commonly used host, accounting for more than 70% of proteins produced for scientific research, and 50% of commercially manufactured proteins^19^. In concordance with many other studies on cytoplasmic expression of human recombinant proteins, expression of OSM in *E. coli* largely results in the accumulation of the protein in inclusion bodies, impeding downstream purification due to the high cost and time-consuming optimisation for solubilisation, refolding and purification required to prepare bioactive OSM^17^.

Although some research has been carried out on OSM production, there is little information on the soluble expression of OSM in the cytoplasm of *E. coli*. Various solubility enhancement tags such as thioredoxin (Trx), small ubiquitin-related modifier (Sumo), glutathione S-transferase (GST), maltose-binding protein (MBP), N-utilisation substance protein A (Nusa), human protein disulphide isomerase (PDI) and the b‘a’ domain of PDI (PDIb‘a’) have been used to enhance the soluble cytoplasmic expression of recombinant proteins in *E. coli*^20–24^. Additionally, engineering of *E. coli* strains such as Origami and SHuffle that are better able to support disulphide bond formation has proven useful for enhancing the production of large quantities of soluble proteins in the cytoplasm^25,26^. However, to date, no single study has investigated the soluble expression of OSM using solubility enhancement tags. Herein, OSM was fused with hexahistidine (His6) and other fusion tags, and cytoplasmic expression and solubility in BL21(DE3) were tested. In addition, soluble expression of His6-OSM was shown to be higher in Origami 2 and SHuffle strains than in BL21(DE3). An efficient purification protocol for the production of OSM in soluble form in *E. coli* was also established, and bioactivity of the protein was confirmed by *in vitro* inhibition of Th17 cell differentiation.

## Results

### Construction of eight vectors for expression of OSM fusions

**Figure 1:**
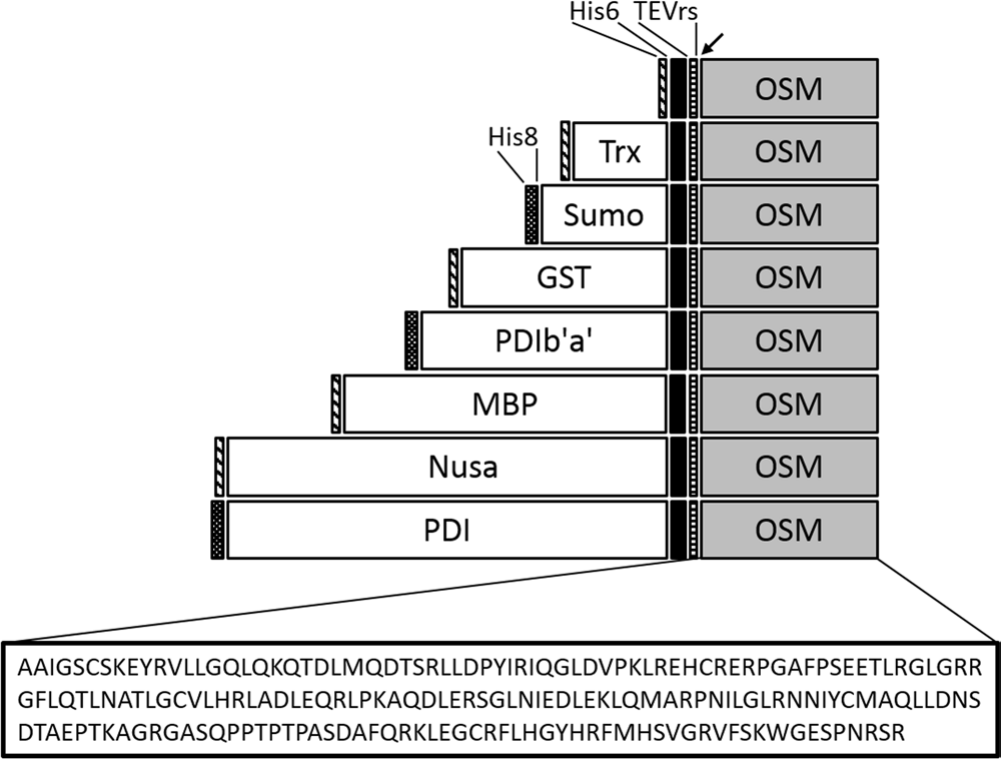
Schematic representation of the domain structure of the OSM fusion variants. The eight OSM fusion variants were designed using the Gateway cloning method. His6, hexa(poly)histidine; Trx, thioredoxin; Sumo, small ubiquitin-related modifier; GST, glutathione-S-transferase; PDIb‘a’, b‘a’ domain of full-length human PDI; MBP, maltose-binding protein; Nusa, N-utilisation substance protein A; PDI, full-length human PDI. His6 tag was fused to the N-terminus of Trx, GST, MBP and Nusa; His8 tag was fused to the N-terminus of Sumo, PDIb′a′ and PDI. Fusion protein expression is driven by the IPTG-inducible T7 promoter, with ampicillin as the selection marker. The arrow indicates the TEV protease cleavage site.

To determine whether OSM could be expressed in soluble form in *E. coli*, eight plasmids for expressing N-terminal His6, Trx, Sumo, GST, MBP, Nusa, PDI and PDIb‘a’-tagged OSM variants were successfully prepared using Gateway BP and LR recombination reactions. To obtain tag-free OSM, a tobacco etch virus protease recognition site (TEVrs) was placed between each tag and the OSM-encoding sequence. All constructs also contained His6/His8 tags to facilitate affinity purification (Fig. 1). They were transformed into BL21(DE3), SHuffle or Origami 2 host strains, and protein expression was tested.

### Evaluation of the expression and solubility of fusion proteins

**Figure 2:**
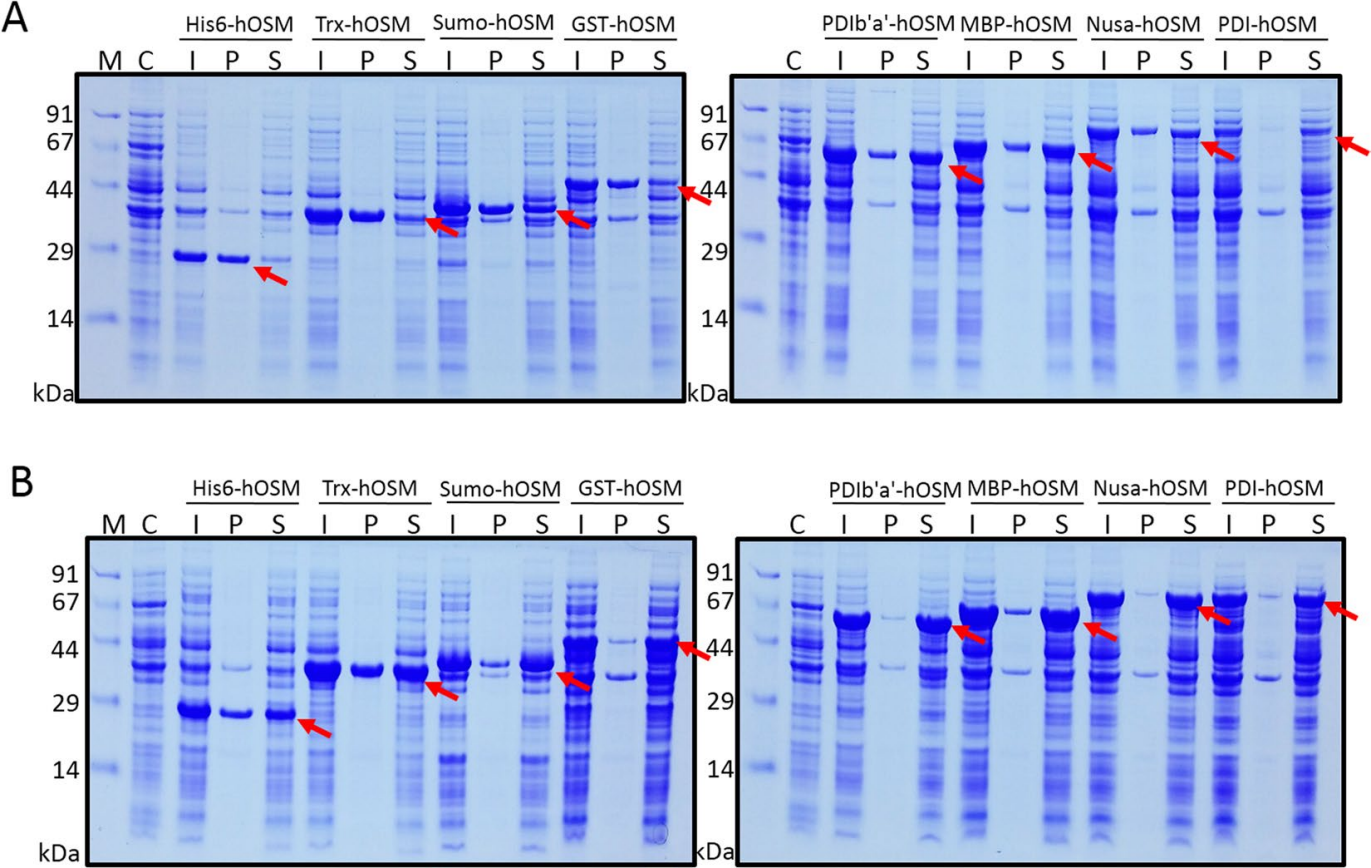
Expression of the eight OSM fusion proteins in BL21(DE3) host cells. Expression of fusion proteins was induced with 0.5 mM IPTG at 37 °C (**A**) or 18 °C (**B**). M, molecular weight markers; C, total cell protein before IPTG induction as a negative control; I, total cell protein after IPTG induction; P, pellet fraction after cell sonication; S, soluble fraction after cell sonication.

**Figure 3:**
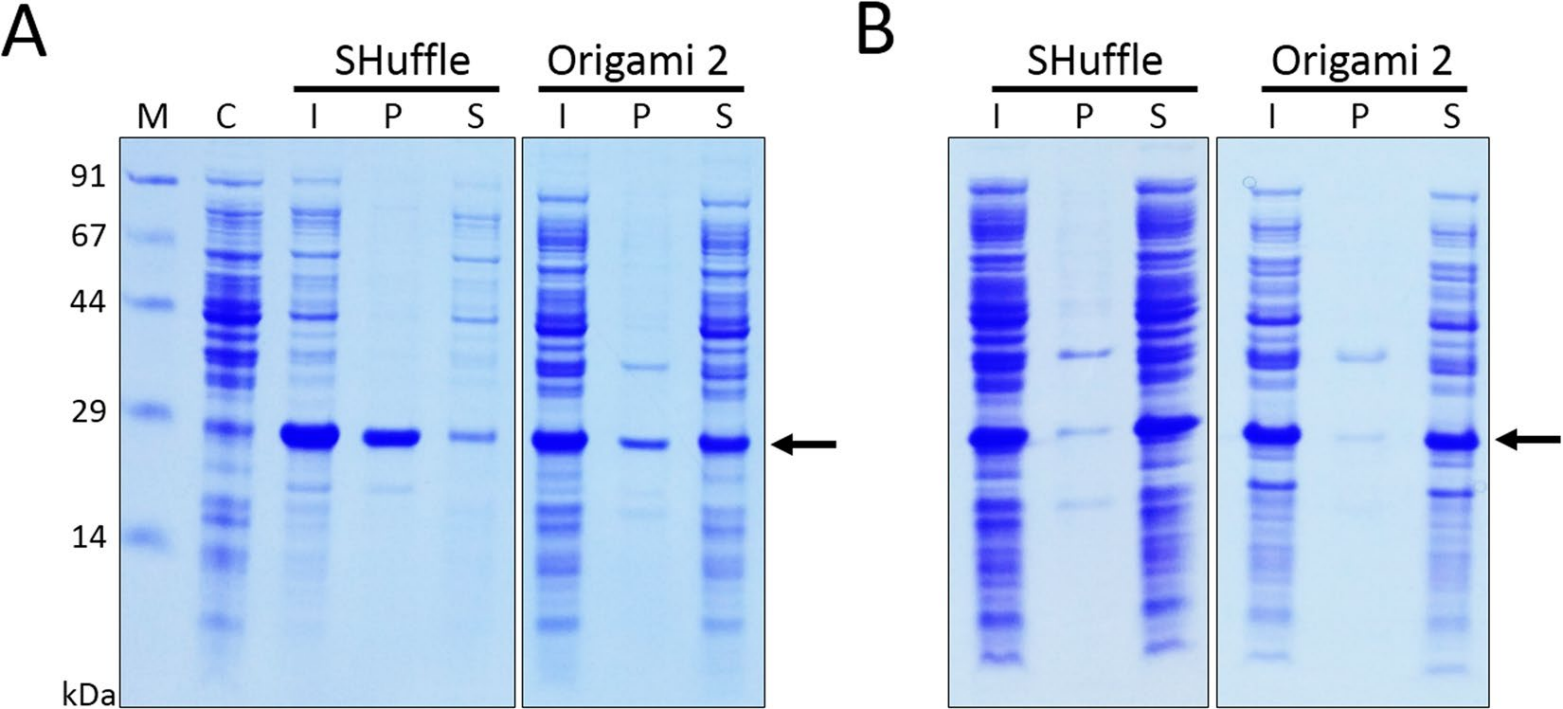
Expression of His6-OSM in SHuffle and Origami 2 host cells. Expression of fusion proteins was induced with 0.5 mM IPTG at 37 °C (**A**) or 18 °C (**B**). M, molecular weight markers; C, total cell protein before IPTG induction as a negative control; I, total cell protein after IPTG induction; P, pellet fraction after cell sonication; S, soluble fraction after cell sonication.

**Figure 4:**
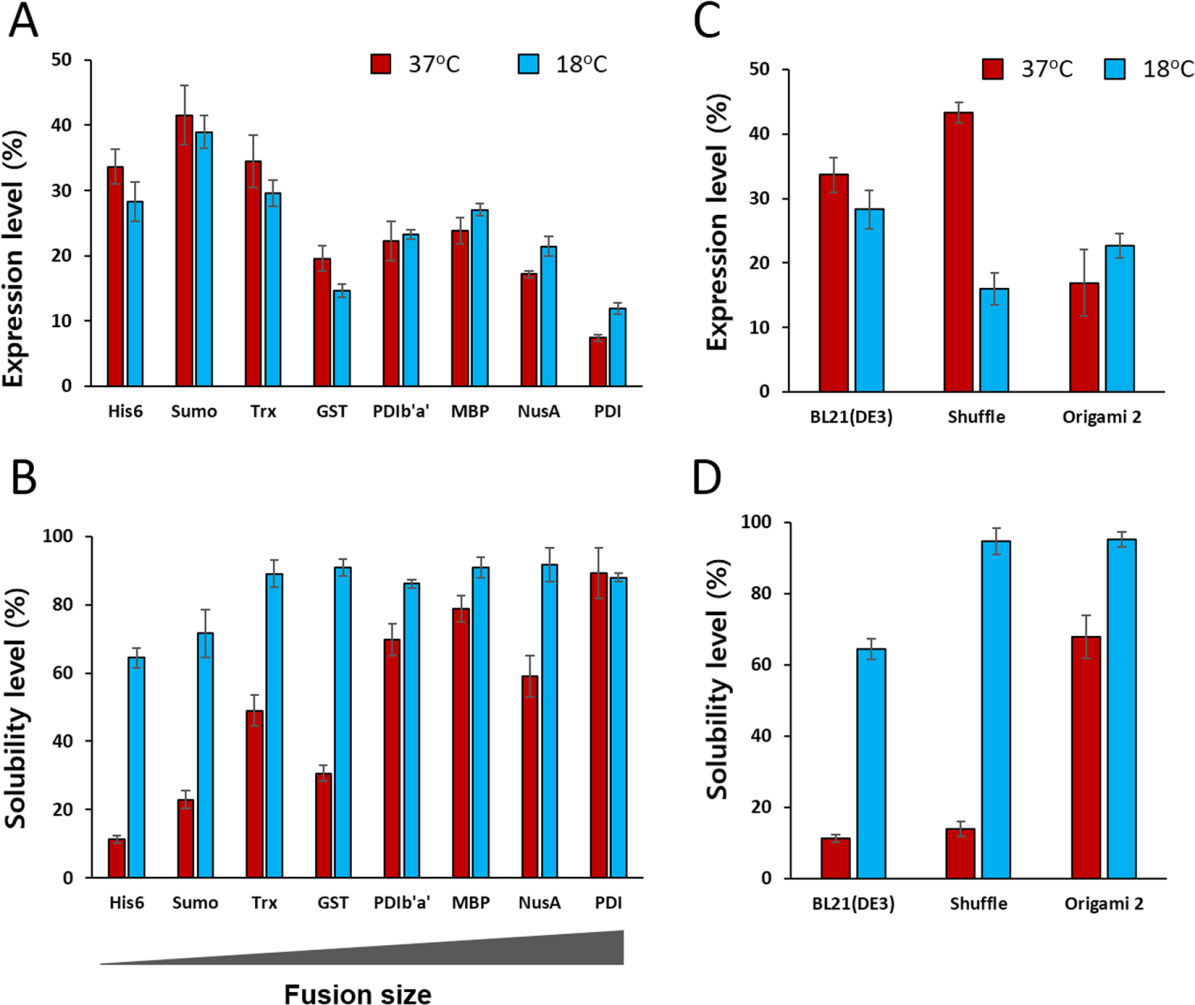
Expression and solubility of OSM fusion proteins in different *E. coli* hosts. (**A**) Expression and (**B**) solubility of OSM fusion proteins in BL21(DE3) cells. (**C**) Expression and (**D**) solubility of His6-OSM expressed in three different *E. coli* host strains. Protein expression and solubility were analysed using densitometry from duplicate experiments. The expression level (%) of the fusion protein was calculated based on the density ratio of target fusion protein vs. total *E. coli* proteins. The solubility level (%) was calculated based on the density ratio of soluble fusion protein to total fusion protein.

Because the expression of OSM fusions was under the control of the T7 promoter, 1-thio-β-d-galactopyranoside (IPTG) was used to induce protein expression from all constructs at both 37 °C and 18 °C. As expected, the expression and solubility of recombinant proteins were varied depending on the fusion tag, induction temperature and expression strain (Figs 2 and 3). To enable comparison of expression and solubility levels among different conditions, protein bands on SDS-PAGE gels were analysed using ImageJ software, and the results are shown in Fig. 4.

When expressed in BL21(DE3) host cells, the expression level of OSM at 37 °C was higher for constructs fused with smaller tags (His6, Sumo and Trx) than those fused with larger tags (GST, PDIb‘a’, MBP, Nusa and PDI; Figs 2 and 4A). However, except for the GST tag, all larger tags increased the solubility of OSM to over 60% at the higher induction temperature. In addition, lowering the induction temperature to 18 °C did not increase the expression level by a significant extent, but it did significantly improve the solubility of all fusion proteins except those with a PDI tag (Figs 2 and 4B). Among the fusion variants tested, the MBP-OSM construct was chosen for further study because this fusion protein displayed high expression and solubility at both induction temperatures, and because the MBP tag can facilitate straightforward downstream purification.

To test whether other host strains could enhance the expression and solubility of OSM, the His6-OSM expression plasmid was introduced into SHuffle and Origami 2 *E. coli* strains. As shown in Fig. 3, overexpression of proteins was achieved with both strains, and the proportion in the soluble fraction was increased when the temperature was decreased from 37 °C to 18 °C. At 37 °C, expression of His6-OSM was higher in SHuffle and BL21(DE3) hosts than in Origami 2 cells, but decreasing the induction temperature increased expression of the fusion protein in Origami 2 cells (Fig. 4C). As expected, induction at 18 °C enhanced the solubility of the fusion by more than 95% in the case of SHuffle and Origami 2 cells, and solubility was also 1.5 times higher in BL21(DE3) cells (Fig. 4D). Based on the impressive expression and solubilisation, the Origami 2 cells were selected for further experiments.

### Purification of OSM from MBP-OSM fusion protein expressed in BL21(DE3) cells

**Figure 5:**
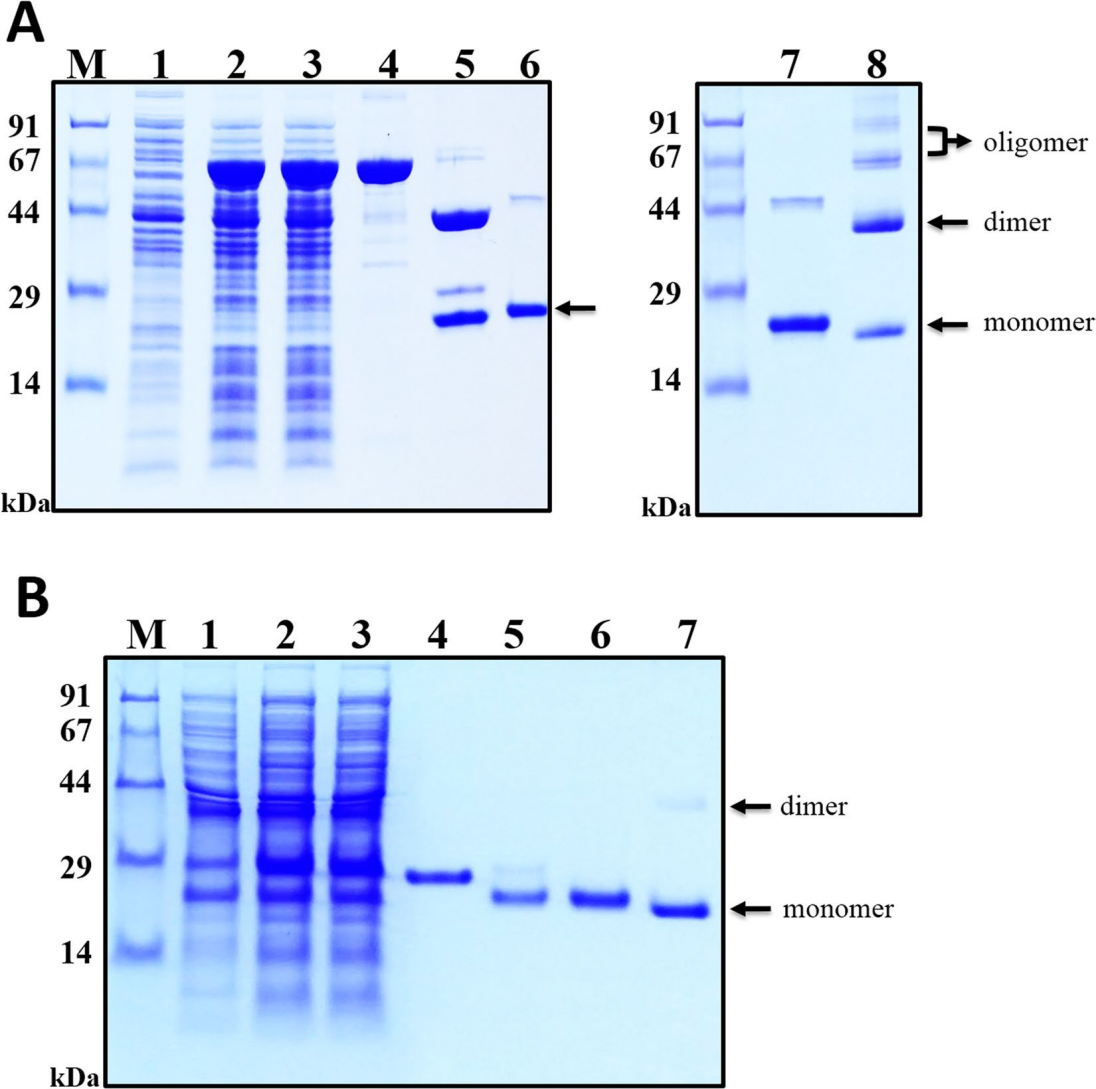
SDS-PAGE analysis of OSM purification from solubly expressed MBP-OSM and His6-OSM in *E. coli*. (**A**) MBP fusion-derived OSM purified from BL21(DE3) cells by IMAC. M, molecular weight markers; lane 1, total cell extract before IPTG induction as a negative control; lane 2, total cell extract with IPTG induction; lane 3, soluble fraction after cell sonication; lane 4, MBP-OSM fusion protein (65.95 kDa) purified by IMAC; lane 5, MBP tag cleavage reaction mixture: TEV protease (28.6 kDa), MBP tag (43.9 kDa) and OSM (22.05 kDa); lane 6, IMAC purification of OSM after TEV cleavage; lanes 7 and 8, the final OSM product under reducing and non-reducing conditions, respectively. (**B**) His6 fusion-derived OSM purified from Origami 2 cells by IMAC. M, molecular weight markers; lane 1, total cell extract before IPTG induction (a negative control); lane 2, total cell extract with IPTG induction; lane 3, soluble fraction after cell sonication; lane 4, His6-OSM fusion protein (25.65 kDa) purified by IMAC; lane 5, His6 tag cleavage reaction mixture: TEV protease (28.6 kDa), His6 tag (3.6 kDa) and OSM (22.05 kDa); lanes 6 and 7, the final OSM product under reducing and non-reducing conditions, respectively. Arrows indicate the positions of OSM monomers (22.05 kDa), dimers (44.1 kDa) and oligomers (≥66.15 kDa).

During the course of protein purification experiments, immobilised metal ion affinity chromatography (IMAC) was performed twice: first, to obtain the MBP-OSM fusion, and then to purify tag-free OSM after TEV protease digestion. After cell disruption, the cell lysate was centrifuged and filtered to achieve a clear supernatant. As shown in Fig. 5A (lane 3), a prominent protein band of ~66 kDa corresponding to MBP-OSM was evident on the gel, and there was no distinct difference between total cell and supernatant fractions, indicating that the fusion protein was highly stable and remained soluble in the sonication buffer. To separate MBP-OSM from *E. coli* proteins and the rest of the cell contents, the supernatant was passed through an IMAC column, and extensive washing was performed to remove most of the impurities, while the fusion protein remained bound due to the high affinity of the His6 tag. SDS-PAGE confirmed that the fusion protein was efficiently purified, and the eluate consisted mainly of a single band corresponding to MBP-OSM, representing a purity of ~89% (Fig. 5A, lane 4; Table 1).

**Table 1:** Purification of OSM from MBP-OSM expressed in BL21(DE3) cells at 18 °C.

Purification step	Total protein (mg)	Purity (%)^a^	OSM (mg)^b^	Yield (%)
Bacterial culture (500 mL)	2,500 (pellet)	—	—	—
Supernatant	155.7	29.5	15.4	100
1^st^ IMAC eluate	51.2	89	15.2	98.7
2^nd^ IMAC eluate	11.6	95	11.02	71.6

^a^Purity of the protein of interest was determined by densitometry using ImageJ.

^b^The amount of OSM was calculated based on the purity of the protein of interest and the size correlation between OSM and the corresponding fusion protein.

Subsequently, the purified MBP-OSM fusion was treated with TEV protease to facilitate tag removal. Since a high salt concentration could inhibit TEV activity^27^, the NaCl concentration in the eluate was reduced to 100 mM to facilitate the tag cleavage reaction. SDS-PAGE revealed that most of the MBP-OSM was cleaved by TEV protease after an overnight incubation at room temperature (Fig. 5A, lane 5). The cleaved sample was then subjected to a second round of IMAC purification. Although the tag-free OSM does not include a His6 tag, the protein was still able to bind to the column, presumably through non-specific interactions, but it was easily eluted by including 50 mM imidazole in the buffer (Fig. 5A, lane 6). Owing to the presence of a His6 tag, the MBP portion of the fusion protein, any undigested fusion protein and TEV protease were only removed from the column by adding buffer containing a higher concentration of imidazole. Further analysis by ImageJ showed that the purity of the final tag-free OSM was ~95%, and 11.02 mg OSM was prepared from a 500 mL culture, representing a recovery yield of ~71.6% (Table 1). After endotoxin removal, the endotoxin level in the final purified OSM was lower than 0.2 EU/μg, which is still below the threshold range (<1 EU/µg) required for a safe protein product. In addition, the SDS-PAGE results presented in Fig. 5 (lane 6 and 7) showed that the final OSM product was mainly a single monomer band in reducing conditions (22.05 kDa), but a mixture of monomer, dimer (44.1 kDa) and other oligomers ($\ge$ 66.15 kDa) in non-reducing conditions.

### Purification of OSM from the His6-OSM fusion expressed in Origami 2 cells

Purification of His6 fusion-derived OSM was conducted according to the procedure described above for the purification of OSM from the MBP fusion, but with slight modifications in buffer constituents. Briefly, 2 M urea was added to the purification buffer in order to favour binding of His6-OSM to the IMAC column. As shown in Fig. 5B (lane 3), the presence of urea did not significantly alter the solubility, and the fusion protein mostly remained in the supernatant fraction of the cell lysate. A band of ~25.65 kDa corresponding to His6-OSM showed that the fusion protein was successfully purified from the IMAC column with a high degree of purity (~99%; Fig. 5B, lane 4; Table 2). Immediately before the TEV protease cleavage of the fusion protein, urea and NaCl were diluted to a final concentration of 1 M and 125 mM, respectively. As can be seen in Fig. 5B (lane 5), a clear shift in the mobility of the protein band occurred, suggesting that tag removal was almost complete. After the second round of IMAC, a final yield of 3.27 mg OSM with high purity was achieved from a 500 mL culture, representing a recovery yield of 18% (Table 2). The final endotoxin level in the sample was measured and was below the threshold range (<1 EU/µg). Under non-reducing conditions, the His6 fusion-derived OSM was predominantly monomeric, but a small amount of protein corresponding to the dimer form was also detected.

**Table 2:** Purification of OSM from His6-OSM expressed in Origami 2 cells at 18 °C.

Purification step	Total protein (mg)	Purity (%)^a^	OSM (mg)^b^	Yield (%)
Bacterial culture (500 mL)	1,420 (pellet)	—	—	—
Supernatant	100.5	21.8	18.8	100
1^st^ IMAC eluate	14.2	99	12.08	64.3
2^nd^ IMAC eluate	3.3	99	3.27	18

^a^Purity of the protein of interest was determined by densitometry using ImageJ.

^b^The amount of OSM was calculated based on the purity of the protein of interest and the size correlation between OSM and the corresponding fusion protein.

### Characterisation of purified OSMs

**Figure 6:**
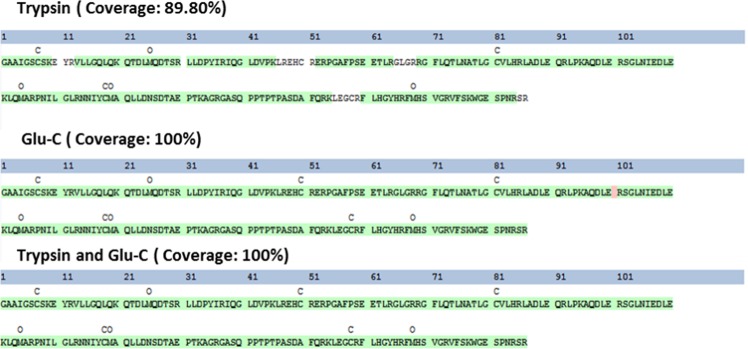
LC-MS/MS sequencing of peptides for protein identification was performed using two enzyme-digested samples (trypsin and Glu-C). The percent coverage for protein identification is presented by displaying the identified peptides against the whole protein sequence. Trypsin = 89.80%, Glu-C = 100% and both enzymes = 100%. The confidence of each peptide sequence is colour-coded based on percolator confidence scores (green, high confidence, p-value < 0.01; red, low confidence, p-value ≥ 0.01).

To confirm the identity of the purified OSM proteins, liquid chromatography tandem mass spectrometry (LC-MS/MS) analysis of two enzyme-digested samples (trypsin and Glu-C) was performed. As shown in Fig. 6, based on a multi-consensus identification approach, OSM was unambiguously identified, with 100% sequence coverage. Further, we identified one correct intra-chain disulphide bond between residues C7 and C128 in the monomeric fraction of His6 fusion-derived OSM. No peaks representing the disulphide bond pattern of the MBP fusion-derived OSM were detected in any sample (Supplementary Fig. S5). Additionally, DTNB assay showed that the free sulfhydryl groups in His6 and MBP fusion-derived OSMs were 5.31 ± 0.68 and 1.3 ± 0.14 (n = 3), respectively. CD spectra of the two OSMs were also analysed to estimate secondary structure of the proteins (Supplementary Fig. S7). Analysis using CD multivariate SSE program demonstrated that His6 and MBP fusion-derived OSMs have similar secondary structures with 24% α-helixes, 26% β-sheets, and 13% turns.

### Purified OSM proteins inhibit Th17 cell differentiation

**Figure 7:**
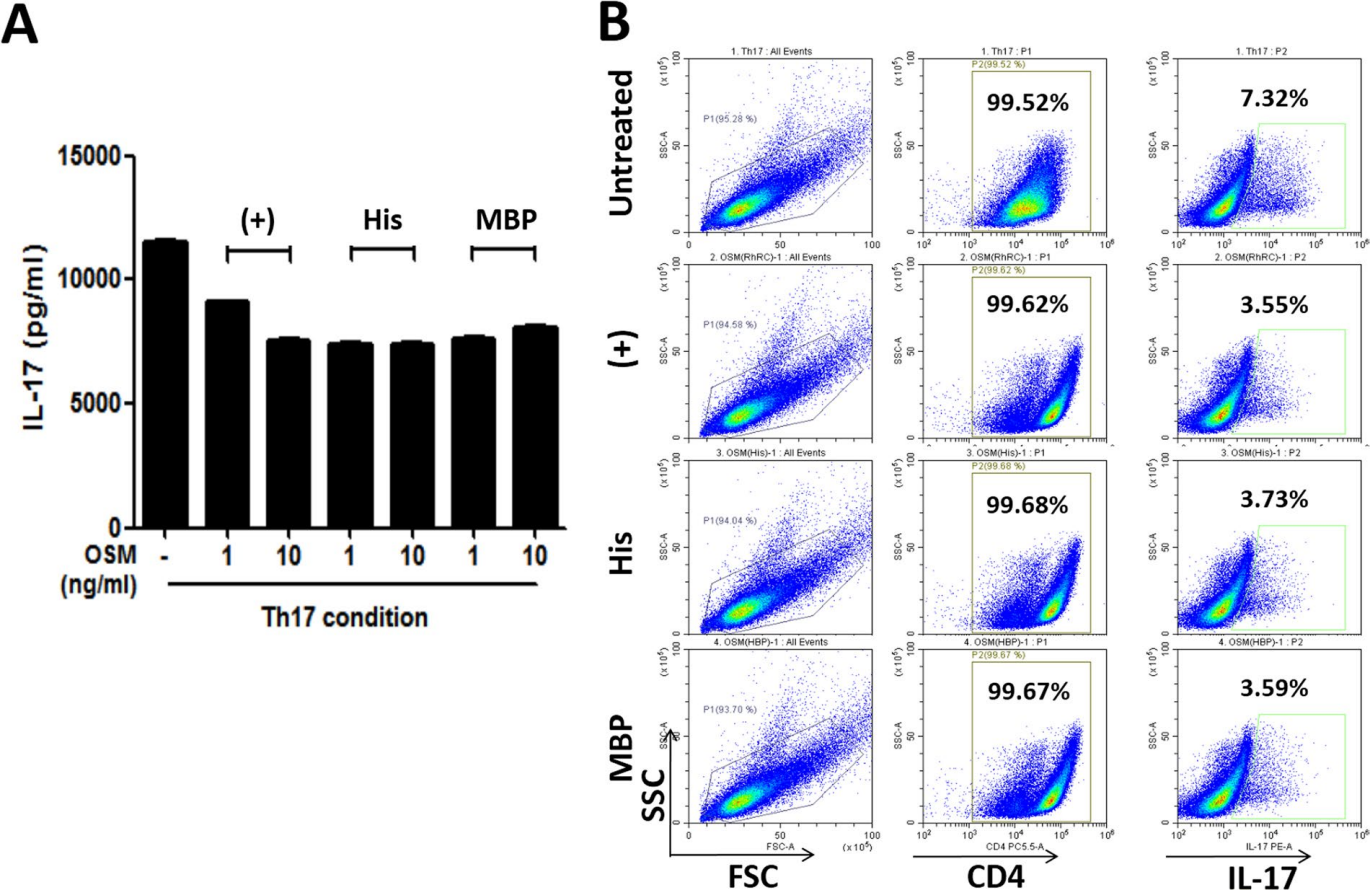
Inhibitory effect of the commercial and purified OSM proteins on the differentiation of CD4^+^ T cells into Th17 cells. CD4^+^ T cells were treated with commercial or purified OSMs under Th17 cell-polarising conditions (n = 3). After 72 h, the culture supernatants were analysed by ELISA to determine the levels of secreted IL-17 (**A**), and the cells treated with 10 ng/mL OSM proteins were analysed by flow cytometry to determine the intracellular levels of IL-17 in differentiated CD4^+^ T cells (**B**). (+), commercial OSM; His, OSM purified from His-OSM; MBP, OSM purified from MBP-OSM.

OSM has an inhibitory effect on the differentiation of CD4^+^ T cells into Th17 cells^28^. To determine the activity of the purified OSM proteins, CD4^+^ T cells were isolated and treated with commercial or purified OSM proteins under culture conditions suitable for Th17 cell polarisation. As shown in Fig. 7A, the presence of OSM proteins in the medium dramatically reduced the secretion of IL-17 at OSM concentrations of 1 and 10 ng/mL. Flow cytometry analysis showed that intracellular IL-17 expression was also decreased following treatment with 10 ng/mL of OSM proteins (Fig. 7B). There was no significant difference in the extent of the reduction of IL-17 secretion between commercial OSM and purified OSM from His6 or MBP fusions. These results demonstrated that the OSM proteins purified in this work suppressed the differentiation of CD4^+^ T cells into Th17 cells similarly to commercial OSM.

## Discussion

Owing to its pleiotropic effects, there have been numerous studies on the pathological and physiological functions of OSM. However, contradictory results have been reported, and further studies are needed before we can fully understand the biological relevance of this cytokine. In this study, the aim was to optimise the soluble expression of recombinant OSM in the cytoplasm of *E. coli* and achieve a simple purification of the soluble form. Three fundamental parameters were tested: the fusion tag, the induction temperature and the *E. coli* host. No such extensive study on the recombinant expression of OSM in a prokaryotic expression system has been reported previously. Therefore, to develop a broader picture of OSM production, it is crucial to enhance our understanding of the soluble expression and purification of OSM in *E. coli*.

As demonstrated in many studies, overexpression of human proteins in *E. coli* often results in their incorporation in inclusion bodies, most likely due to protein misfolding and aggregation^29,30^. In agreement with the results of a previous study^17^, the His6-OSM construct produced insoluble OSM following induction at 37 °C (Fig. 2A), presumably due to complications associated with refolding and purification to obtain bioactive protein. To overcome such challenges, solubility-enhancing tag systems have been established, and they provide excellent tools for the highly-soluble expression of proteins in the cytoplasm of prokaryotic hosts^20–24^. In the present study, the MBP-OSM variant was chosen for further study based on its high solubility in BL21(DE3) at both induction temperatures, which was increased dramatically by 79% at 37 °C and 91% at 18 °C compared with the His6-OSM fusion protein, representing a 7- and 1.4-fold increase, respectively (Fig. 4A,B). One possible explanation for this might be that MBP possesses intrinsic chaperone activity^31^, allowing it to bind to hydrophobic residues of OSM and promote proper protein folding, resulting in increased solubility of MBP-tagged OSM.

The induction temperature is also a key factor affecting the solubility of target proteins. There is evidence to suggest that lowering the induction temperature increases the expression and activity of host chaperones, which in turn lowers or even eliminates protein aggregation, and facilitates the soluble expression and correct folding of recombinant target proteins expressed in *E. coli*^32^. The results of the present study were consistent with this hypothesis; decreasing the induction temperature generally increased the solubility of all tested constructs. In particular, the solubility of smaller fusions with His6, Sumo, Trx or GST tags was increased ~2-fold when expressed at 18 °C. However, low temperature cultivation can decrease the growth rate of the *E. coli*^33^ and different induction temperatures are probably more suitable for different recombinant target proteins, thus an optimisation step should be performed.

The *E. coli* cytoplasm is a reducing environment that does not facilitate the formation of the correct disulphide bonds within heterologous proteins. Thus, the production of disulphide-rich proteins in the cytoplasm of prokaryotic host cells does not generally produce high yields of correctly folded, bioactive recombinant proteins^30,32,34^. Many genetically engineered *E. coli* host strains such as Origami and SHuffle have a more oxidative cytoplasmic environment, and they have been employed successfully for production of disulphide-rich heterologous proteins^30,32,34^. Since there are two disulphide bonds present in the OSM protein^35^, these two strains were employed in an attempt to overcome the challenge of poor solubility and disulphide formation associated with expression using the His6-OSM construct in the reducing environment of *E. coli* BL21(DE3) cells. As shown in Fig. 4C,D, the expression and solubility of His6-OSM was markedly affected by both the induction temperature and the host cell type. Interestingly, we found that the soluble expression of His6-OSM was more favoured in Origami 2 and SHuffle strains than in BL21(DE3) at both 37 °C and 18 °C. In addition, to address the question of whether a combination of the two fundamental parameters, the fusion tag and *E. coli* host type, could simultaneously enhance both the expression and solubility of OSM, the MBP-OSM plasmid was also introduced into two mutated *E. coli* strains. In general, unlike the His6 fusion, no significant improvement in the expression and solubility of MBP-OSM was observed in Origami 2 or SHuffle strains at either temperature (Supplementary Fig. S1). Hence, under the conditions tested, the combination of these two factors is not required for soluble expression of OSM in the cytoplasm. These findings also suggest that the MBP tag is fundamentally responsible for promoting the expression and solubility of the OSM fusion protein, whereas the oxidative cytoplasmic environment is critical for the His6-OSM expression in mutated strains.

As shown in Fig. 5, dimeric and higher-order oligomeric forms of MBP-OSM expressed in BL21(DE3) cells and of His6-OSM expressed in the Origami 2 strain were observed. Furthermore, as may be seen on the gel filtration chromatograms of the two OSM sources, two major peaks corresponding to dimers/oligomers and monomeric fractions were detected, with relative retention volumes of approximately 55 mL and 66 mL, respectively (Supplementary Fig. S2). In addition, based on the height and area of the observed peaks, and SDS-PAGE analysis, OSM produced from MBP-OSM in BL21(DE3) tended to oligomerise to a greater extent than that produced from His6-OSM in Origami 2 strain. In human, OSM contains two native intra-chain disulphide bonds (between residues C50–C168 and C7–C128) and one free cysteine (C81)^35^. Hence, to determine the pattern of disulphide bond linkage in the proteins, the purified proteins were subjected to MALDI-TOF/TOF MS and DTNB assay. A correct intra-chain disulphide bond between residues C7–C128 was observed in the monomeric fraction of His6 fusion-derived OSM (Supplementary Fig. S5), and a wrong disulphide bond between residues C81–C128 was detected in both monomeric and dimeric forms of protein prepared from the His6 fusion (Supplementary Figs S5 and S6). However, DTNB result revealed that five free sulfhydryl groups present in OSMs purified from His6. This could be explained that the detected disulphide bonds from mass spectra were formed due to air oxidation of the free cysteine residues from incompletely alkylated fragments. In case of MBP-derived OSM, there was only one free sulfhydryl group estimated from DTNB assay and the free cysteine, C81 was indicated in all mass spectra (Supplementary Figs S3 and S4). Thus, it would appear that the two correct disulphide bonds present in the protein which could not be detected by the mass analysis. The CD analysis produced similar combinations of the secondary structures between the OSMs from MBP-OSM and His6-OSM (Figure S7). However, the result is different from that of the previous report^36^. We don’t have an explanation for this discrepancy. Nonetheless, the proteins exhibited good activity, similar to that of the commercial OSM, in a CD4^+^ T cell assay (Fig. 7). This suggests a possibility that the final purified OSM proteins have a structure proper for the binding of the receptor and activating the cells.

In the present study, OSM proteins were prepared using two purification strategies, both of which utilised TEV protease cleavage to remove the fusion tag, which is a very common method for successful tag removal that has been applied in numerous studies^20–24^. This cleavage step has been shown to be important since low efficiency at this stage can hamper the downstream purification of target proteins^25,37^. As shown in Fig. 5, the TEV cleavage was highly efficient for removing the tag and generating tag-free OSM from the fusion protein, demonstrating that TEV protease was successfully incorporated into the purification strategy without detriment to the final yield. Another important advantage of the chosen purification strategy was the inclusion of a His6 tag attached to the N-terminus of both MBP- and His6-OSM fusions to facilitate the straightforward purification of the target protein before and after tag removal steps. However, in standard buffer conditions, the binding capacity of both fusion proteins to the IMAC column did not reach 100%, resulting in some protein losses during purification. Triton X-100 is a typical non-ionic surfactant, and urea is a widely used chaotropic agent; both reagents are frequently employed to facilitate reversible protein denaturation^38,39^. When added to the purification buffer at concentrations of 0.5% and 2 M, Triton X-100 and urea acted as mild denaturants to assist the exposure of His6 tags, allowing them to efficiently bind to the IMAC column (Fig. 5A,B, lane 4). A previous report showed that a His6-OSM fusion protein was insolubly expressed in BL21(DE3) cells with 2.3 mg OSM was obtained from 1 L cells following refolding^17^. This yield is considerably lower than that achieved in the current study, in which 3.27 mg OSM was successfully purified in soluble form from only 0.5 L cells. In a follow-up study using a yeast expression host, 6.94 g OSM was produced from an 80 L fermentation, representing a yield of 62%^14^. As shown in Table 1, even though *E. coli* culture conditions were not optimised, 11.02 mg OSM was obtained from 0.5 L cells, with a final yield of 71.6% that is appreciably higher than that achieved in the aforementioned study. OSM is a multifunctional cytokine, and a better understanding of its functions requires further investigation. The final OSM proteins produced using the two strategies adopted in this study exhibited a similar inhibitory effect on differentiation of CD4^+^ T cells into Th17 cells to that of commercially *E. coli*-derived OSM (Fig. 7). This suggests that the *E. coli*-derived OSM may be applicable not only for inflammatory research, but also for pathological and physiological studies.

In conclusion, the soluble expression of OSM was successfully achieved by testing various solubility enhancement tags and different *E. coli* host strains. Both the expression and solubility of OSM varied with fusion tag, induction temperature and *E. coli* host. Following initial testing, a novel prokaryotic production strategy was established for the efficient production of bioactive OSM in soluble form. Previous research focusing on the prokaryotic production of OSM is scarce, hence these findings represent a significant step forward in the soluble production of OSM in *E. coli*, and provide a method for soluble OSM production that will benefit fundamental research and potential therapeutic applications.

## Materials and Methods

### Materials

All purchased chemicals were of analytical grade. Ampicillin was acquired from Duchefa Biochemie (Haarlem, Netherlands). Lambda integrase and excisionase were from Elpis Biotech (Daejeon, Korea). Dialysis membranes were from Viskase (Darien, IL). Dithiothreitol (DTT) and 1-thio-β-d-galactopyranoside (IPTG) were from Anaspec (Fremont, CA). Coomassie brilliant blue R-250 and TRIS were purchased from Amresco (Solon, Ohio). Imidazole was from Daejung Chemicals (Siheung, Korea). l-Cysteine, NaCl and glycerol were purchased from Samchun Chemical (Pyongtaek, Korea). 2-mercaptoethanol was from Yakuri Pure Chemicals (Kyoto, Japan). All columns used for protein purification were purchased from GE Healthcare (Piscataway, NJ). The Limulus Amebocyte Lysate test kit was from Lonza (Basel, Switzerland). *E. coli* BL21(DE3), SHuffle were from New England Biolabs (Ipswich, MA) and Origami 2(DE3) was acquired from Novagen (Madison, WI). Amicon Ultra-15 Centrifugal Filter Units were from Millipore (Billerica, MA). 3-(4,5-Dimethylthiazol-2-yl)-2,5-diphenyltetrazolium (MTT), ExtrAvidin-alkaline phosphatase, phorbol 12-myristate 13-acetate (PMA), 5,5′-dithiobis-(2-nitrobenzoic acid) (DTNB), and ionomycin were from Sigma Aldrich (St. Louis, MO). Commercial OSM was purchased from R&D Systems (Minneapolis, MN). Dulbecco’s Modified Eagle’s Medium (DMEM), 0.25% trypsin-EDTA, foetal bovine serum (FBS) and penicillin-streptomycin were from GIBCO (Carlsbad, CA). Anti-CD3 mAb, anti-CD28 mAb and GolgiStop were from BD Pharmingen (San Jose, CA). Anti-IFN-γ, anti-IL-4, anti-IL-2, anti-IL-17, biotinylated anti-IL-17, TGF-β, IL-6, IL-17 and recombinant mouse OSM were purchased from R&D Systems (Minneapolis, MN). CD4-Peridinin Chlorophyll Protein Complex (PerCP), anti-mouse IL-17-Phycoerythrin (PE) and CytoPerm/CytoFix were from BD Biosciences (San Diego, CA).

### Construction of expression plasmids

The Gateway cloning system was used to design OSM expression vectors. BP and LR recombination reactions were accomplished as previously described^21,24^. To facilitate expression of OSM in *E. coli*, a codon-optimised oligonucleotide encoding the tobacco etch virus protease recognition site (TEVrs), ENLYFQ/G, followed by a gene encoding the 195 amino acid residue mature OSM protein (NCBI Reference Sequence: CAG30420.1) was synthesised (GenScript, Piscataway, NJ) and inserted into the pDONR207 vector via a BP reaction to generate the entry clone pENTR-OSM. Accordingly, a series of expression vectors were prepared by LR reactions using pENTR-OSM and the eight destination vectors pDEST-HGWA (His6), pDEST-SUMO (Sumo), pDEST-HXGWA (Trx), pDEST-HGGWA (GST), pDEST-HMGWA (MBP), pDEST-HNGWA (Nusa), pDEST-PDI (PDI) and pDEST-PDIb‘a’ (PDIb‘a’). All expression constructs were confirmed by DNA sequencing (Macrogen, Daejeon, Korea).

### Cytoplasmic expression and solubility testing of fusion variants

*E. coli* BL21(DE3) cells were separately transformed with the eight expression plasmids, and single colonies were inoculated into Luria-Bertani (LB) medium containing 50 μg/mL ampicillin and cultured at 37 °C overnight. Overnight cultures were transferred into fresh LB medium containing ampicillin at a 1:100 v/v ratio, and cells were cultured at 37 °C with shaking at 200 rpm. Protein expression was induced with 0.5 mM IPTG when the OD_600_ value reached 0.5–0.7, and culturing was continued at 37 °C for 5 h or 18 °C for 18 h. Cells were collected by centrifugation and protein expression was analysed by SDS-PAGE using 10% tricine gels.

To test the expression of OSM in different *E. coli* hosts, the His6-OSM plasmid was introduced into genetically engineered Origami 2 and SHuffle strains. Protein expression was conducted as described above for BL21(DE3) cells. Expression of His6-OSM in different expression hosts was also analysed by SDS-PAGE using 10% tricine gels.

### Purification of OSM from MBP-OSM expressed in BL21(DE3) cells

After an 18 h induction at 18 °C with 0.5 mM IPTG, the 500 mL culture expressing MBP-OSM was harvested by centrifugation at 3,800 × g for 30 min at 4 °C, and cell pellets were used immediately or stored at −80 °C until needed. For cell lysate preparation, frozen cells were resuspended in 100 mL buffer A (20 mM TRIS-HCl pH 8.0, 5% glycerol v/v, 500 mM NaCl) containing 0.5% Triton X-100 (v/v) and completely homogenised with a JY99-IIDN ultrasonic cell disruptor (Ningbo Scientz Biotechnology, Guangdong, China). The cell lysate was then centrifuged at 23,000 × g for 30 min at 4 °C to remove cell debris, and the supernatant containing MBP-OSM was filtered through a 0.4 μm membrane prior to purification by immobilised metal ion affinity chromatography (IMAC).

The cleared supernatant containing MBP-OSM was passed through a 20 mL HisPrep FF 16/10 column pre-equilibrated with five column volumes (CVs) of buffer A. The column was then adequately washed with five CVs of buffer A containing 0.5% Triton X-100 (v/v), followed by five CVs of the same buffer but without Triton X-100. To remove any non-specifically bound proteins, the column was washed with more than 10 CVs of buffer A containing 50 mM imidazole. The bound fusion protein was then eluted from the column with five CVs of buffer A containing 500 mM imidazole.

Prior to tag removal using TEV protease, eluate fractions containing fusion protein were mixed with buffer B (20 mM TRIS-HCl pH 8.0, 5% glycerol v/v) to give a final concentration of 100 mM NaCl. Subsequently, the diluted sample was incubated with TEV protease at a ratio of 1:15 (w/w) at room temperature overnight. The cleaved mixture was dialysed against buffer A and Triton X-100 was added to a final concentration of 0.5% (v/v) prior to loading onto the IMAC column pre-equilibrated with buffer A containing 0.5% Triton X-100 (v/v). The column was again washed with five CVs of buffer A containing 0.5% Triton X-100 (v/v) followed by five CVs of the same buffer but without Triton X-100. The bound OSM was eluted from the column by washing with buffer A containing 50 mM imidazole. After this step, MBP tags and TEV protease still bound tightly to the column and were eluted with buffer A containing 1 M imidazole. The eluted OSM was dialysed twice against 1× PBS, pH 7.4 (dilution factor of 100 each time), and stored at −70 °C until needed for further experiments, or freeze-dried for long-term storage. Fractions from all purification steps were checked on 10% tricine SDS-PAGE gels, and protein concentration was measured using the Bradford method with BSA as a standard.

### Purification of OSM from His6-OSM expressed in Origami 2 cells

All purification steps for the preparation of OSM from His6-OSM expressed in Origami 2 were conducted as described above unless otherwise stated. Briefly, cell pellets from 500 mL cultures were resuspended in 50 mL buffer C (20 mM TRIS-HCl, pH 8.0, 5% glycerol v/v, 250 mM NaCl, 2 M urea) and completely homogenised using the same sonication method mentioned above. The cell lysate was then centrifuged and the supernatant containing His6-OSM was used as the crude sample for IMAC.

A 5 mL HisTrap HP column pre-equilibrated with buffer C was used to capture the fusion protein, and non-specifically bound impurities were washed from the column by applying more than 10 CVs of buffer C containing 50 mM imidazole. After washing, the bound fusion protein was eluted from the column with buffer A containing 300 mM imidazole.

Before the cleavage reaction, NaCl and urea were reduced to 125 mM and 1 M, respectively, by mixing with buffer B. TEV protease was added at a ratio of 1:20 (w/w) and the reaction was incubated at room temperature overnight. The cleaved protein mixture was dialysed against buffer D (20 mM TRIS-HCl pH 8.0, 5% glycerol v/v, 250 mM NaCl) and was then purified using a 5 mL IMAC column. The tag-free OSM was eluted from the column by washing with buffer D containing 50 mM imidazole. His6 tags and TEV protease were then removed from the column with buffer A containing 1 M imidazole. The purified OSM was dialysed against 1× PBS pH 7.4 and stored at −70 °C or freeze-dried for long-term storage. Protein samples from all purification steps were visualised on a 10% tricine SDS-PAGE gel, and the Bradford method was used to measure protein concentration.

### SDS-PAGE analysis of protein expression and quantification of protein solubility

A 5× sample buffer (312.5 mM TRIS-HCl pH 6.8, 50% glycerol, 5% SDS, 0.05% bromophenol blue, 100 mM DTT) was prepared for mixing with protein fractions prior to running on 10% tricine SDS-PAGE gels. Protein bands were visualised with Coomassie brilliant blue R-250. Protein expression and solubility and target protein purity were quantified using ImageJ software (http://imagej.nih.gov/ij) as described in previous studies^20–24^.

### Endotoxin removal

Triton X-114 was used to remove endotoxins from purified samples following a previously described method^40^. To measure the endotoxin level, the QCL-1000 Endpoint Chromogenic Limulus Amebocyte Lysate (LAL) Assay kit was used following the manufacturer’s instructions. A 96-well plate was pre-incubated at 37 °C, and 50 μL OSM or protein standards were added to each well and incubated for 10 min with 50 μL LAL. Next, 100 μL chromogenic substrate solution was added and the mixture was incubated at 37 °C for 6 min. To terminate the reaction, 100 μL stop reagent (25% v/v glacial acetic acid) was added, and a UV/vis spectrophotometer was used to measure the absorbance at 405–410 nm.

### Characterisation of the purified OSM proteins by size exclusion chromatography

To assess their oligomeric status, the final proteins purified after cleavage from the His and MBP fusions were injected onto a HiLoad 16/600 Superdex 75 pg gel filtration column. For the experiment, 1× PBS (pH 7.4) was used as the mobile phase, with a flow rate of 1 mL/min. The peaks corresponding to the relative sizes of the proteins of interest were then identified in the chromatograms.

### Mass spectrometry of purified OSM

Protein sample aliquots (100 µg) were reduced and alkylated by DTT and iodoacetamide (IAA), respectively. The treated samples were digested overnight by trypsin (cleaving the peptide bond after arginine and lysine residues, but not before proline) or Glu-C (cleaving the peptide bond after glutamic and aspartic residues). For the identification of OSM proteins, nano-LC-MS/MS experiments were carried out on a NanoACQUITY ultra-performance liquid chromatography (UPLC) system connected to a Q-Exactive spectrometer (Thermo Scientific, Bremen, Germany) through a nanoelectrospray ion source. Database searches (SEQUEST) were conducted by Proteome Discoverer (Thermo Fisher Scientific, ver. 1.4.0.288).

### Disulphide bond identification by MALDI-TOF/TOF MS

The disulphide bond status of the purified OSM proteins was analysed by MALDI-TOF/TOF MS following a previously described protocol^41^. Briefly, to prepare a non-reduced sample, the dimeric and monomeric protein forms (20 µg) were treated with 10 mM iodoacetamide (IAA) and the samples were cleaved by trypsin (1.4 µg) at 37 °C, overnight. For a reduced sample, the proteins were incubated with 10 mM DTT for 10 min at 95 °C prior to IAA treatment and trypsinisation. After desalting and concentrating, by passing through a Ziptip C18 (Millipore), all samples were mixed with the matrix (α-cyano-4-hydroxycinnamic acid). The peptide peaks were identified using MALDI-TOF/TOF™ 5800 (AB SCIEX, Framingham, MA) at the Korea Basic Science Institute (Seoul, Korea).

### Free sulfhydryl quantification by DTNB assay

All prepared solutions were kept on ice and protected from light. Briefly, a 10 mM stock solution of DTNB was prepared in DMSO and diluted with 0.1 M TRIS-HCl, pH 8.0 to make a 0.2 mM working solution. A set of l-Cysteine standard was freshly prepared by serial dilution with 0.1 M TRIS-HCl, pH 8.0. A total of 50 μL of protein samples or l-Cysteine standards was transferred to a 96 well-plate, followed by addition of 50 μL of DTNB working solution. The plate was incubated for 10 min in dark, at 25 °C and the absorbance at 412 nm was measured. Data was expressed as mean ± S.D of at least three experiments.

### Circular dichroism (CD)

CD spectra of monomeric OSMs in 1× PBS (pH 7.4) were measured by using JASCO J-1500 spectropolarimeter (Jasco, Easton, MD). Far-UV CD spectra were recorded from 200 to 250 nm with 0.1-cm path length and a 1-nm bandwidth at 25 °C. The spectrum obtained from buffer alone were used for background subtraction. Tendency of secondary structure of the proteins was estimated by using CD Multivariate SSE program (Jasco).

### CD4^+^ T cell isolation and Th17 cell differentiation

Mouse splenocytes were prepared from the spleens of C57/BL6 mice. Cells were resuspended in 100 μL MACS buffer (1% bovine serum albumin, 5 mM EDTA, 0.01% sodium azide) and incubated with CD4-coated magnetic beads (10 μL/1 × 10^7^ cells) for 10 min at 4 °C. Cells were diluted in 10 μL MACS buffer, centrifuged and resuspended in 500 μL MACS buffer again before magnetic separation in an AutoMACS magnet equipped with a MACS MS column (Miltenyi Biotec, San Diego, CA). The isolated CD4^+^ T cells were then stimulated under Th17 cell-polarising conditions (plate-bound anti-CD3 mAb at 0.5 μg/mL, anti-CD28 mAb at 1 μg/mL, anti-IFN-γ at 2 μg/mL, anti-IL-4 at 2 μg/mL, TGF-β at 2 ng/mL and IL-6 at 20 ng/mL) in the presence or absence of commercial or purified OSMs at 1 or 10 ng/mL for 3 days.

### Enzyme-linked immunosorbent assay

Sandwich ELISA with anti-mouse IL-17 mAb and biotinylated IL-17 polyclonal Ab was used to measure IL-17 in culture supernatants. Briefly, 96-well plates were initially incubated with anti-mouse IL-17 mAb at 4 °C overnight, and wells were treated for 2 h with blocking buffer (PBS containing 1% bovine BSA and 0.05% Tween 20), and then incubated for 2 h with culture supernatants or recombinant IL-17 standards at room temperature. Plates were washed and incubated with biotinylated IL-17 polyclonal Ab for 2 h, followed by an additional 2 h incubation with a 2000-fold dilution of ExtrAvidin-alkaline phosphatase (Sigma Aldrich, St. Louis, MO). Plates were subsequently washed, 50 μL p-nitrophenyl phosphate disodium salt in 1 mg/mL diethanolamine buffer was added to each well and the absorbance at 405 nm was measured using an ELISA microplate reader (Molecular Devices, Sunnyvale, CA).

### Flow cytometry analysis

Differentiated CD4^+^ T cells were stained with anti-mouse CD4-PerCP followed by fixation and permeabilisation with CytoPerm/CytoFix according to the manufacturer’s instructions. Cells were further stained with anti-mouse IL-17-PE and flow cytometry was performed using a FACSCalibur cytometer (BD Biosciences, San Diego, CA).

### Statistical analysis

Statistical analysis was performed using IBM SPSS Statistics 20 for Windows (IBM Corp., Armonk, NY). The statistical significance of differences between multiple groups was evaluated by one-way analysis of variance (ANOVA); when a significant difference was detected, the Bonferroni post-hoc test was used to assess the significance of differences between the individual groups. Values of p < 0.05 were considered to indicate statistical significance. Data are presented as the means ± standard deviation (SD).

## Supplementary information

SUPPLEMENTARY INFO

## Data Availability

All data generated or analysed during this study are included in this published article and its Supplementary Information files.
