# Supplementary material for: Prokaryotic soluble overexpression and purification of oncostatin M using a fusion approach and genetically engineered E. coli strains
Source: Sci Rep. 2019 Sep 23;9:13706. doi: 10.1038/s41598-019-50110-6 (PMC6757106; doi:10.1038/s41598-019-50110-6)
Supplement: Supplementary file 1 — SUPPLEMENTARY INFO [file 41598_2019_50110_MOESM1_ESM.docx]

**Prokaryotic** **soluble overexpression and purification of oncostatin M using a fusion approach and genetically engineered *E. coli* strains**

Minh Tan Nguyen^1^, Musharrat Jahan Prima^1^, Jung-A Song^1^, Julee Kim^1^, Bich Hang Do^1^, Jiwon Yoo^1^, Sangsu Park^1^, Jaepyeong Jang^1^, Sunju Lee^1^, Eunyoung Lee^1^, Michelle de Paula Novais^1^, Hyeon-Beom Seo^2^, Seon-yeong Lee^2^, Mi-La Cho^2^, Chong Jai Kim^3^, Yeon Jin Jang^1^ and Han Choe^1,*^

^1^Department of Physiology, Asan-Minnesota Institute for Innovating Transplantation, Bio-Medical Institute of Technology, University of Ulsan College of Medicine, Asan Medical Center, Seoul 05505, Korea

^2^The Rheumatism Research Center, The Laboratory of Immune Network, CRCID, The Catholic University of Korea, Seoul 06591, Korea

^3^Department of Pathology, Asan-Minnesota Institute for Innovating Transplantation, Asan Medical Center, University of Ulsan College of Medicine, Seoul 05505, Korea

^*^Corresponding author: Han Choe, Phone: +82-2-3010-4292, E-mail: hchoe@ulsan.ac.kr


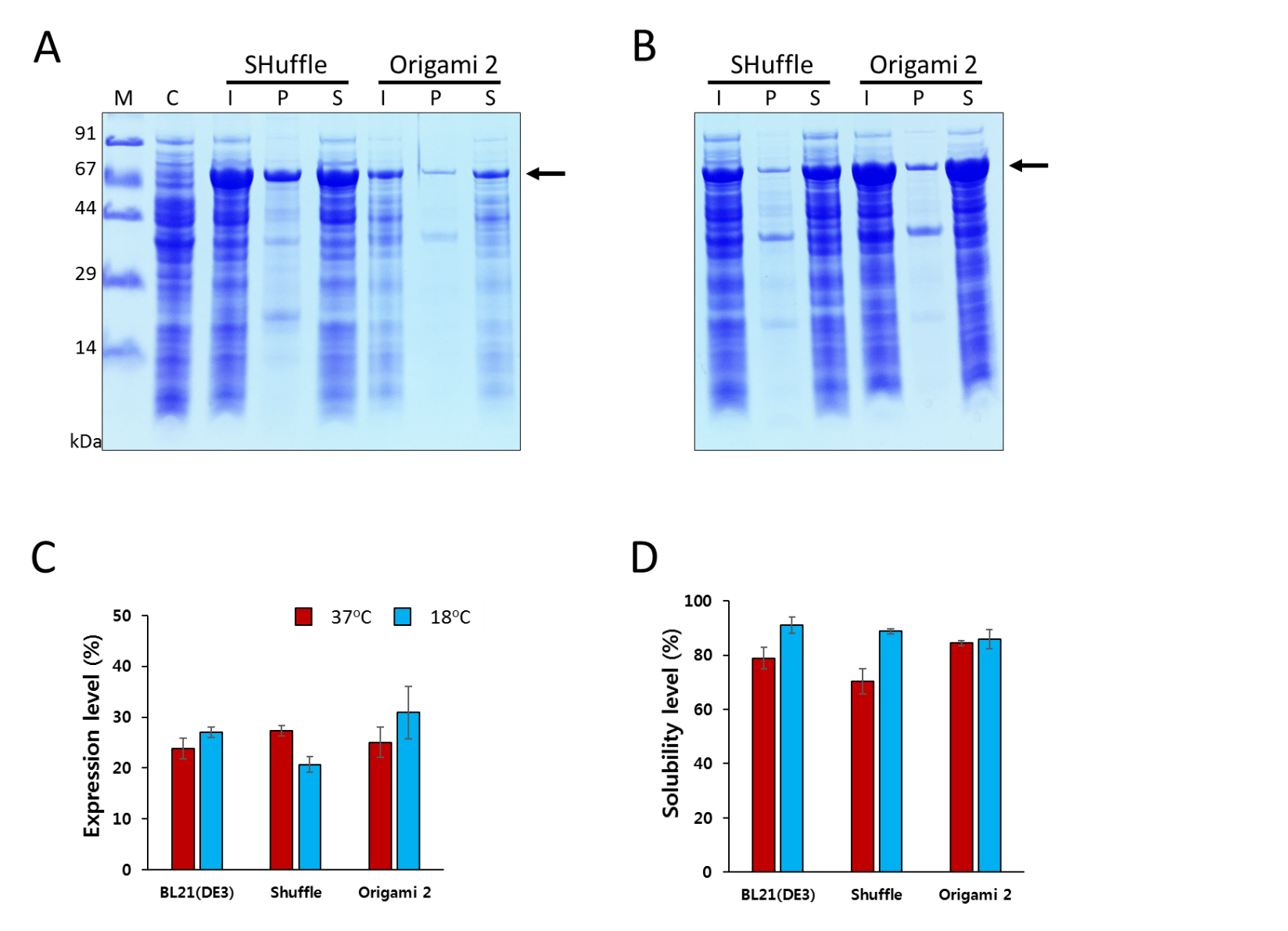


**Supplementary Figure S1.** Expression and solubility of MBP-OSM in the SHuffle and Origami 2 cells. The expression of fusion proteins was induced by the addition of 0.5 mM IPTG at 37°C (A) or 18°C (B). M, molecular weight markers; C, total cell protein before IPTG induction as a negative control; I, total cell protein after IPTG induction; P, the pellet fraction after cell sonication; S, the soluble fraction after cell sonication. The expression (C) and solubility (D) of MBP-OSM were analysed in duplicate experiments using densitometry. The expression (%) of the fusion protein was calculated based on the density ratio of the target fusion protein to total E. coli proteins. The solubility (%) was calculated based on the density ratio of soluble fusion protein to total fusion protein.


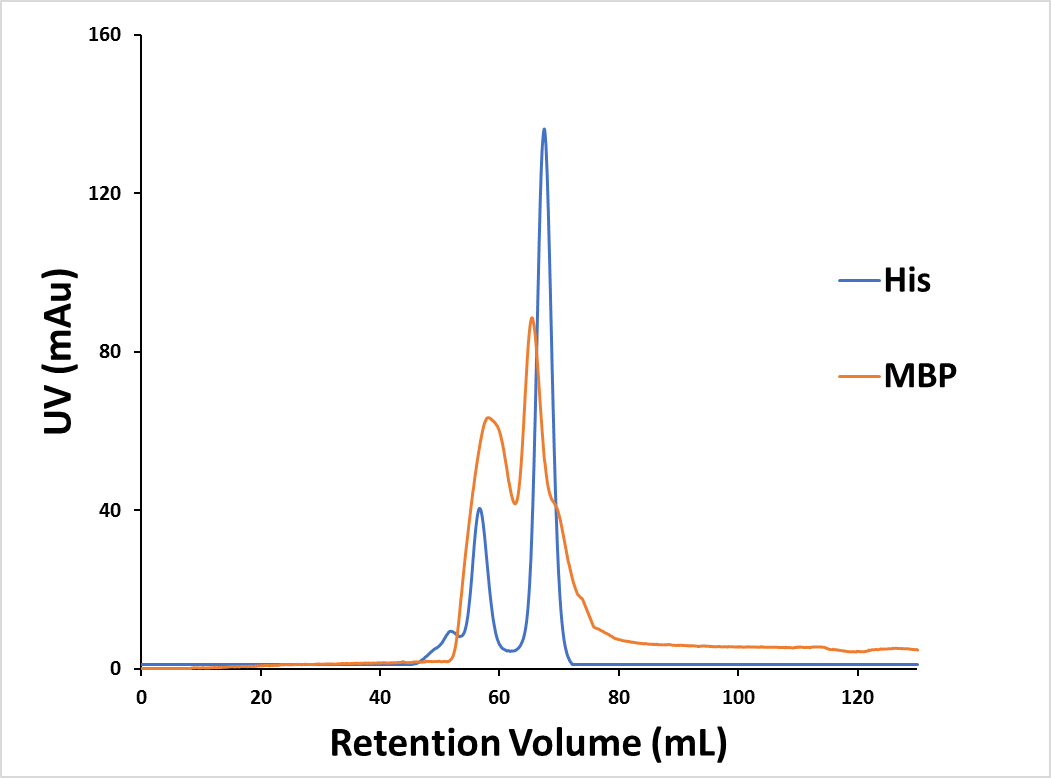


**Supplementary Figure S2.** Chromatogram of the analysis of purified OSM proteins on the HiLoad 16/600 Superdex 75 pg gel filtration column. His, OSM purified from the His-OSM fusion; MBP, OSM purified from the MBP-OSM fusion.


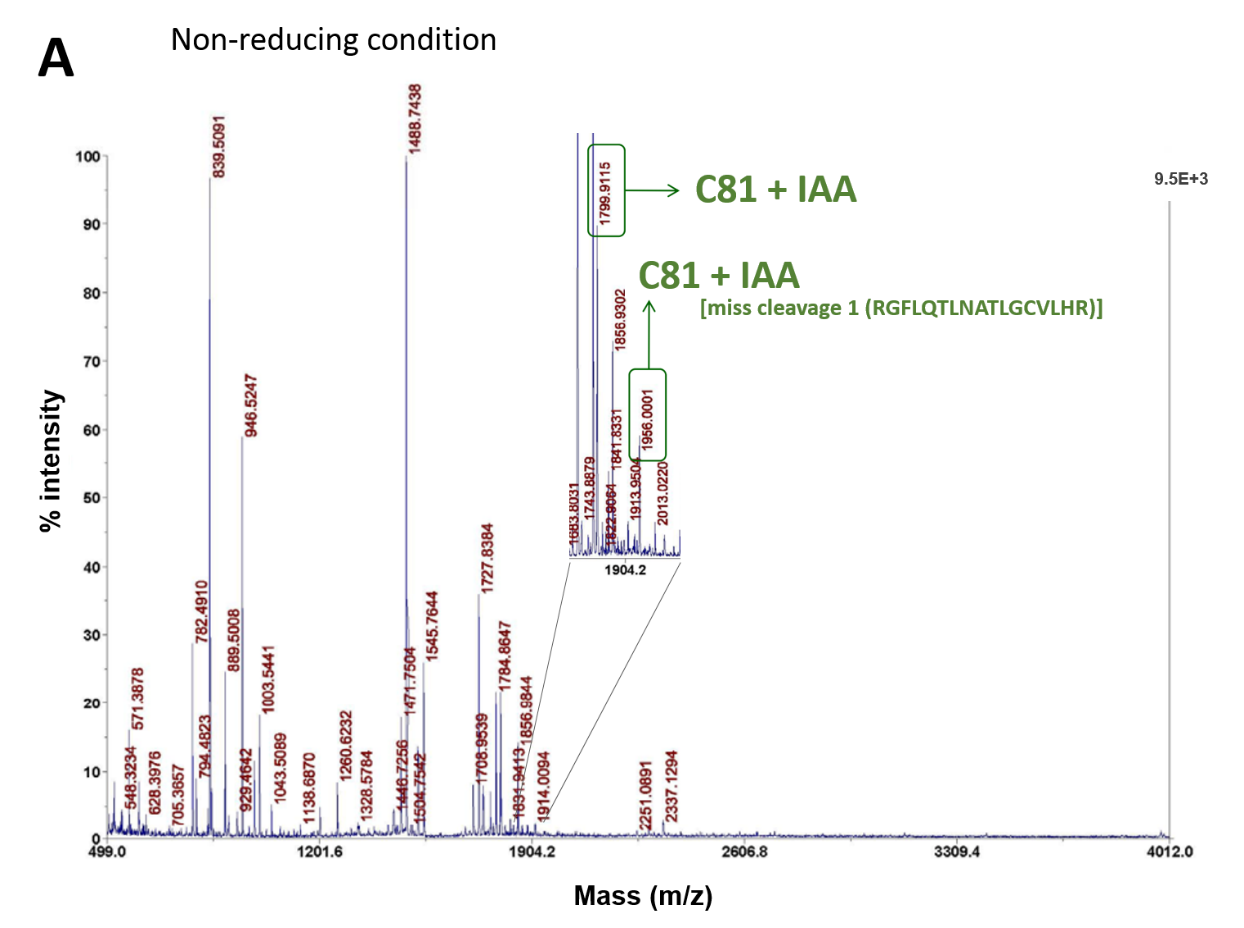

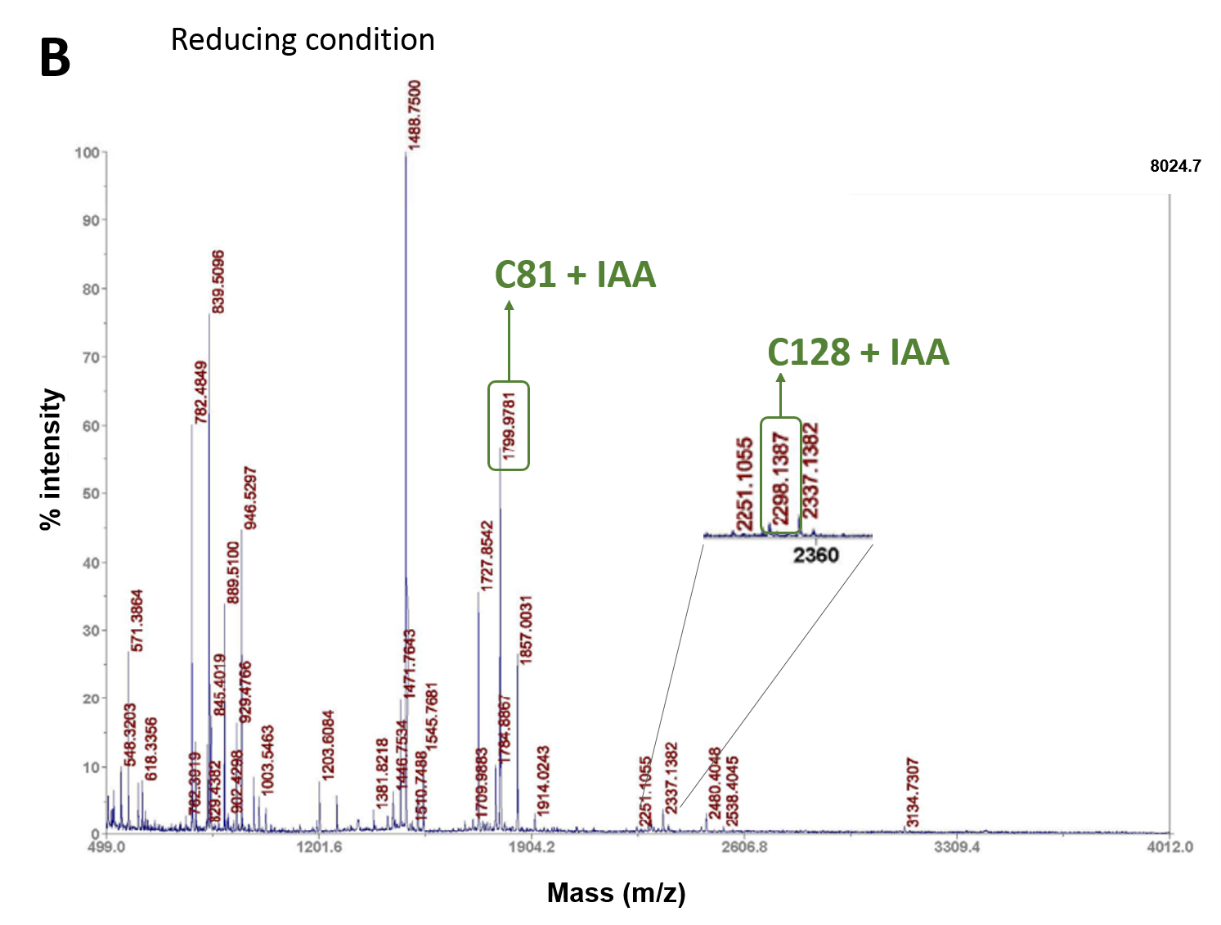


**Supplementary Figure S3.** Disulphide bond identification in MBP fusion-derived OSM (monomer fraction) under non-reducing (A) and reducing conditions (B).


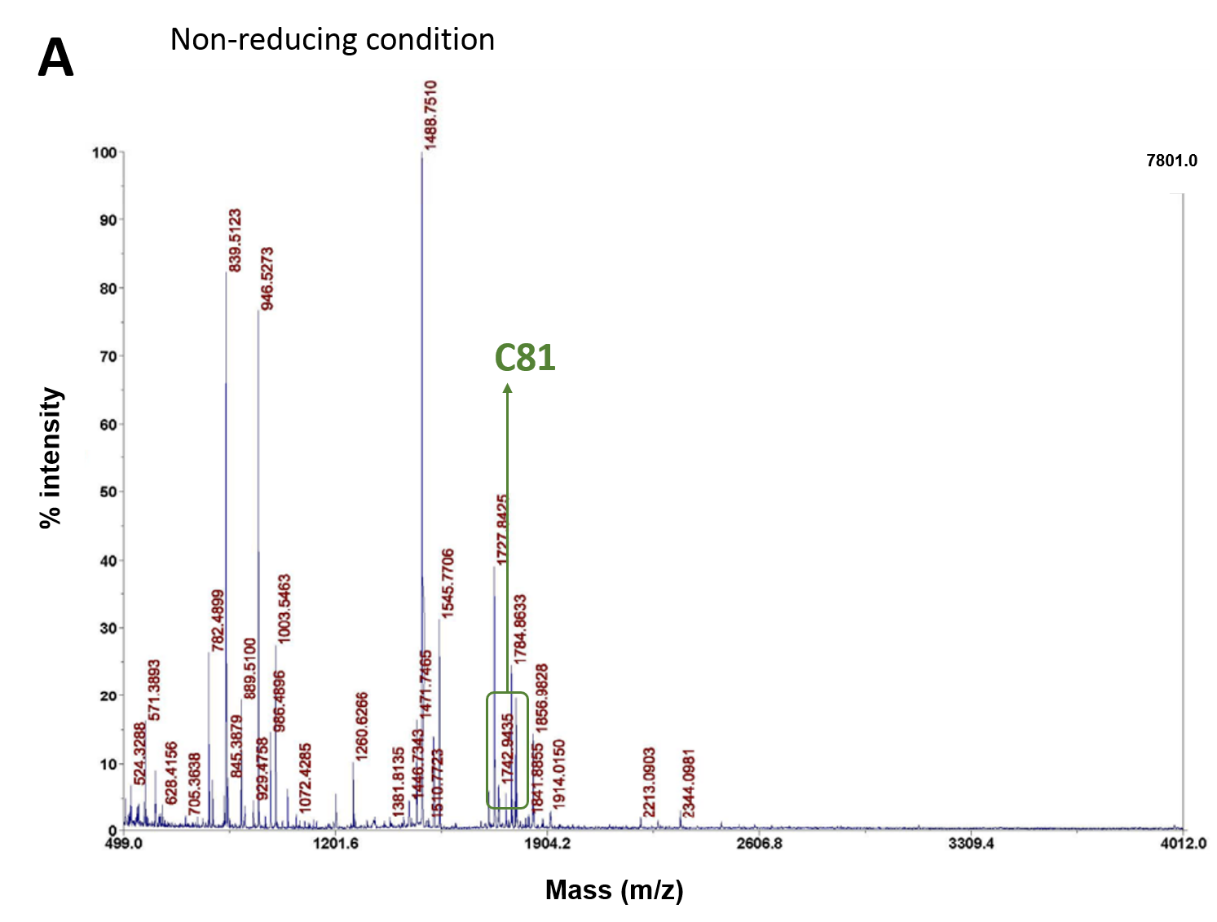


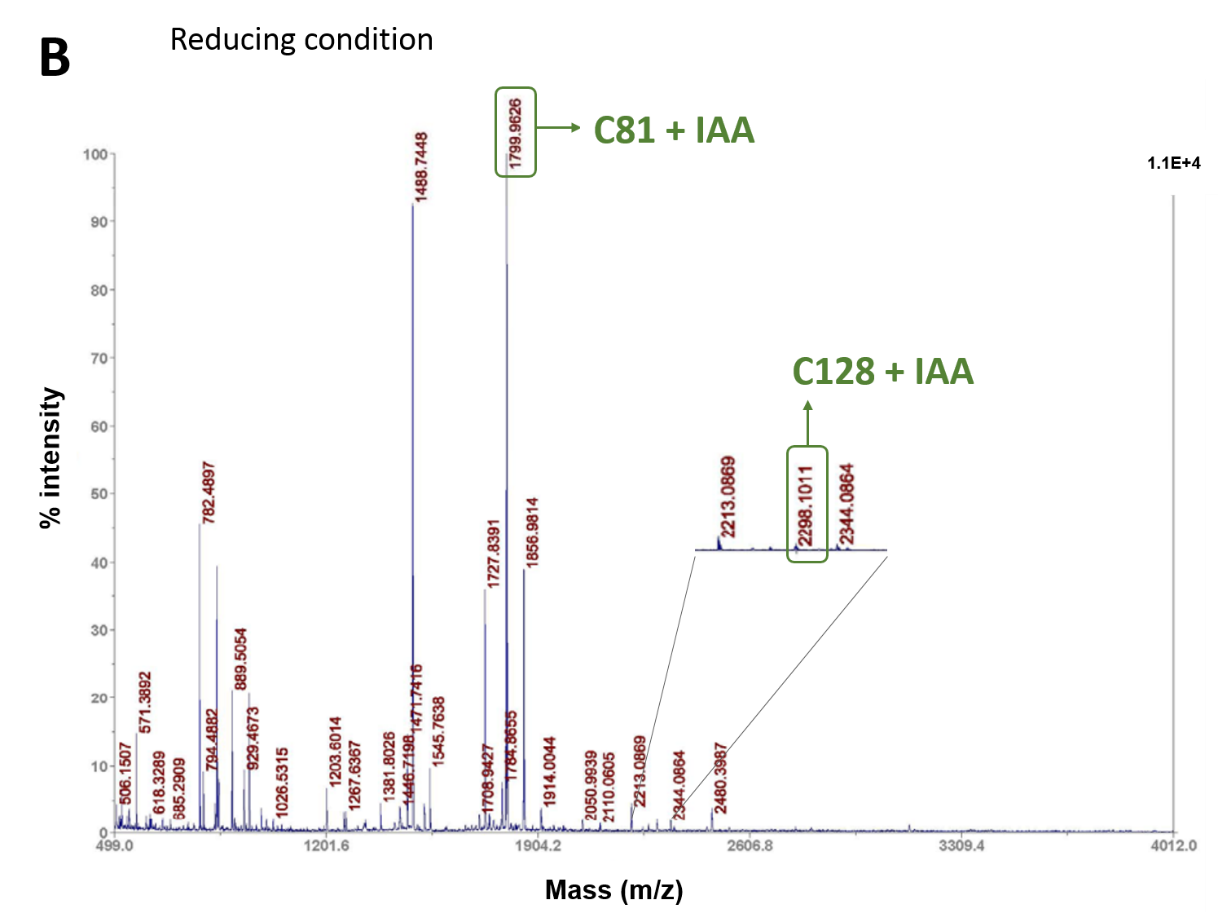
**Supplementary Figure S4.** Disulphide bond identification in MBP fusion-derived OSM (dimer fraction) under non-reducing (A) and reducing conditions (B).


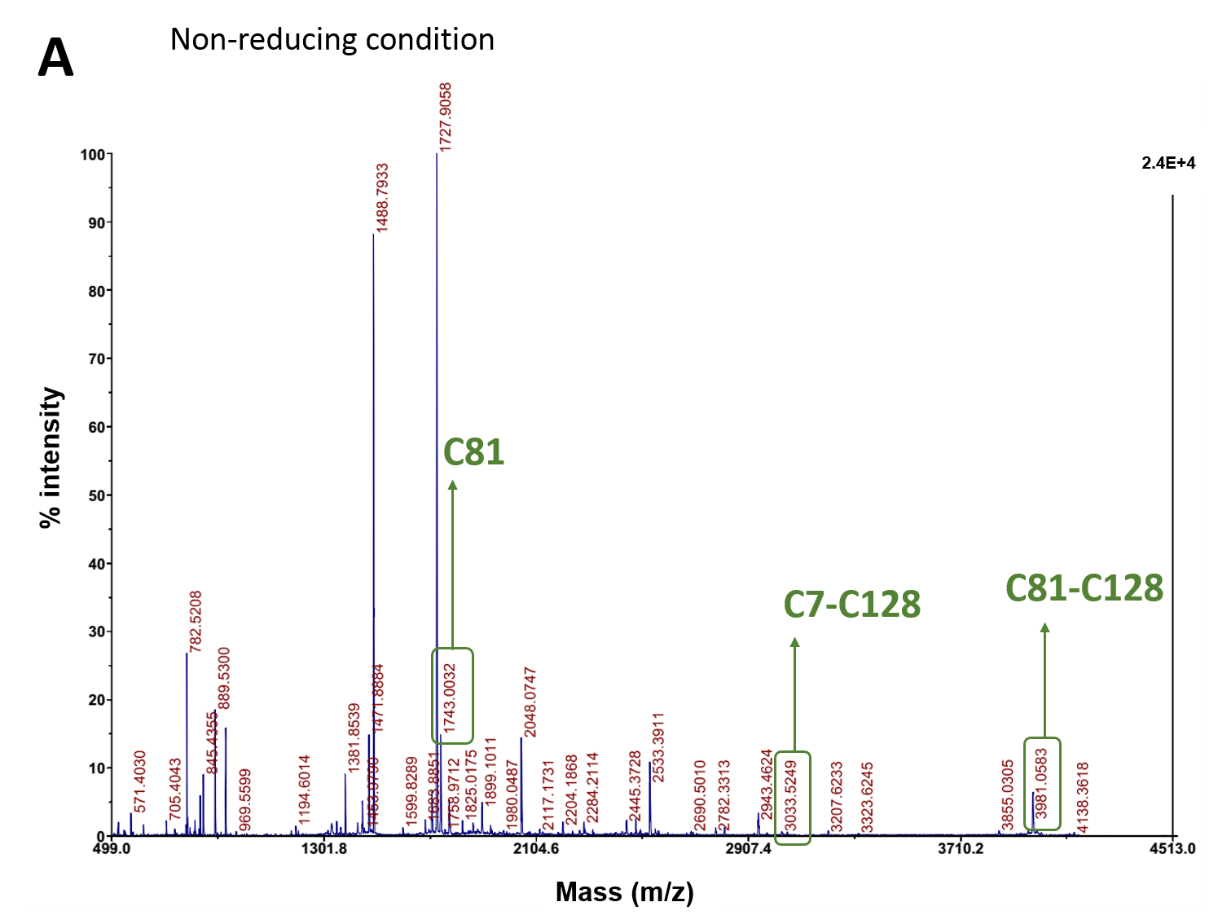


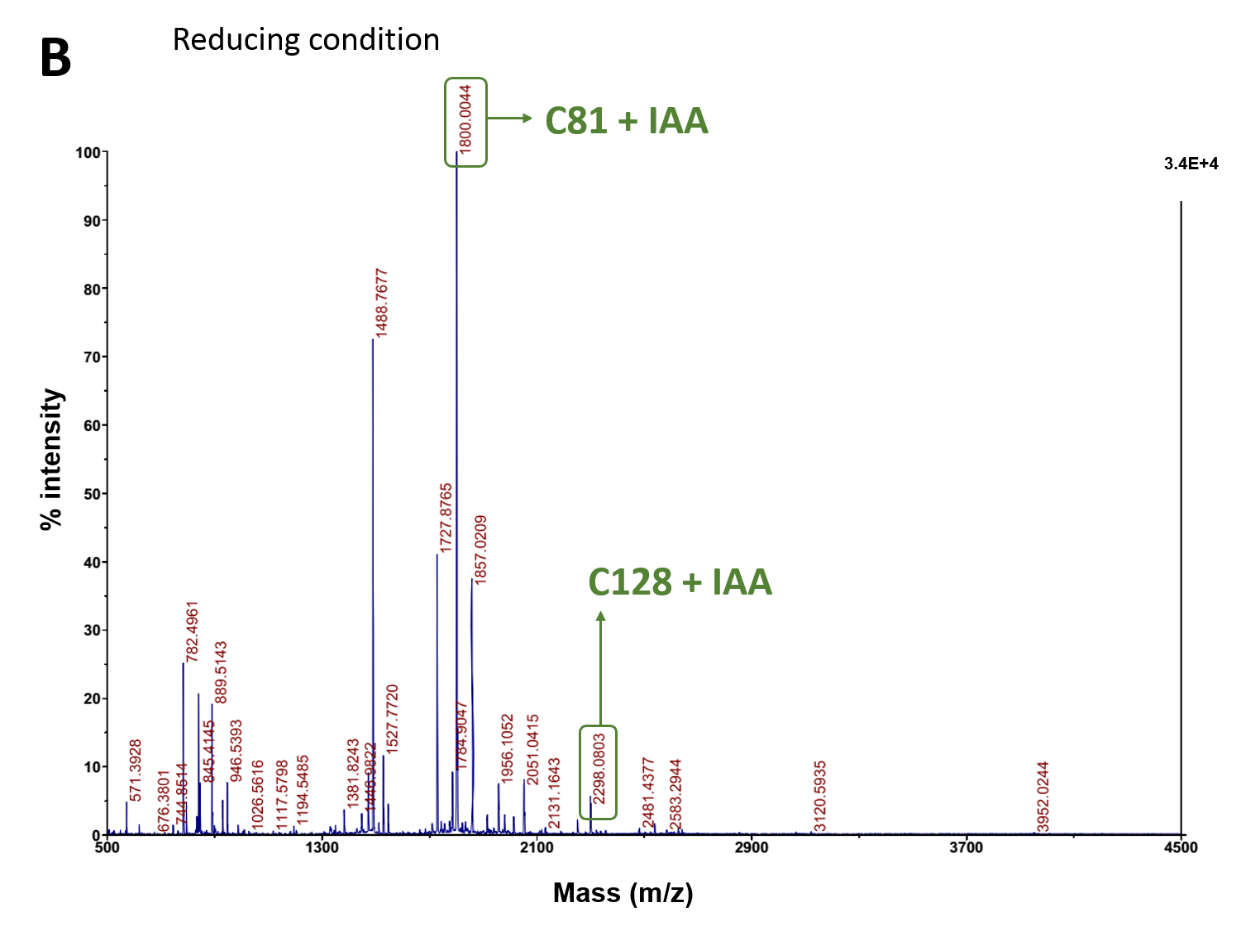


**Supplementary Figure S5.** Disulphide bond identification in His6 fusion-derived OSM (monomer fraction) under non-reducing (A) and reducing conditions (B).


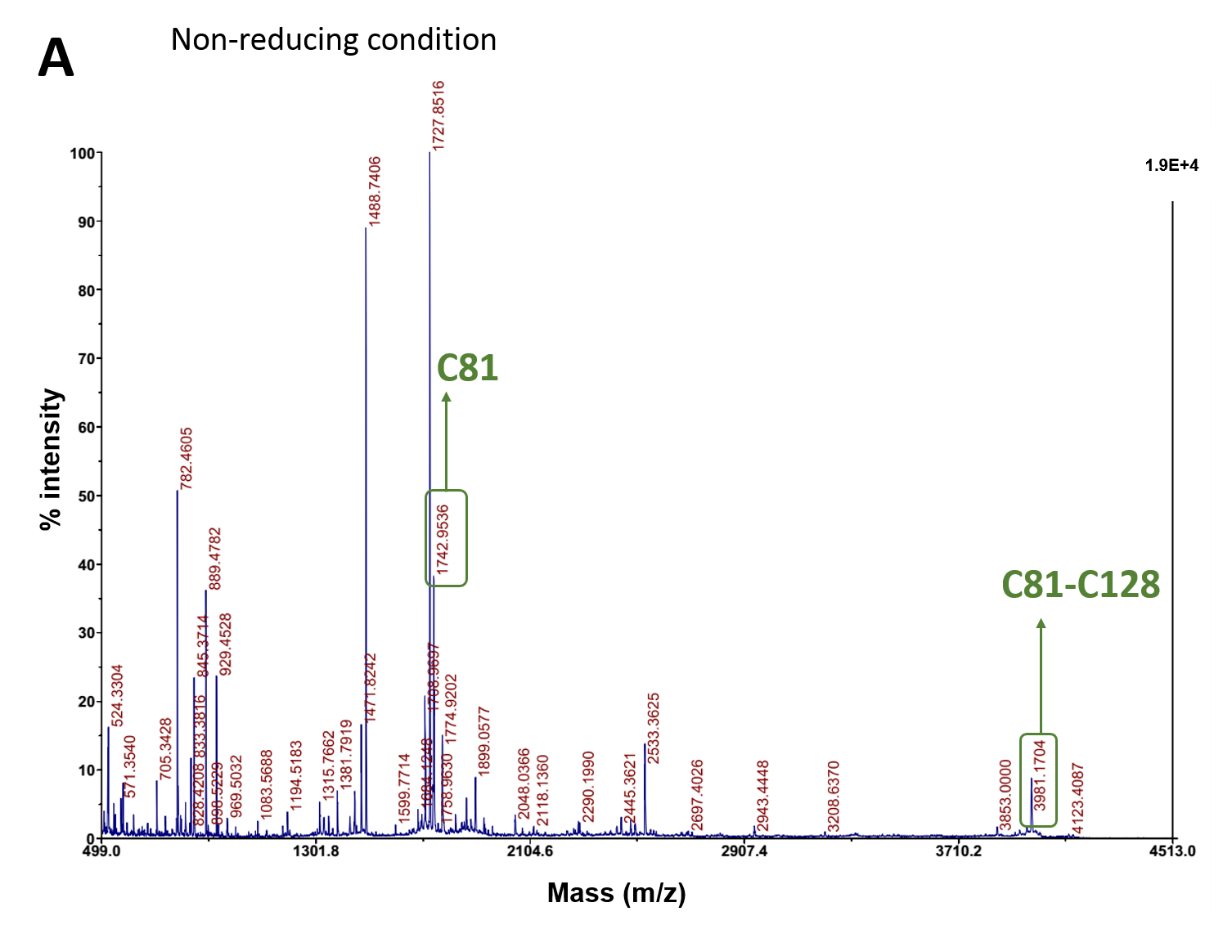

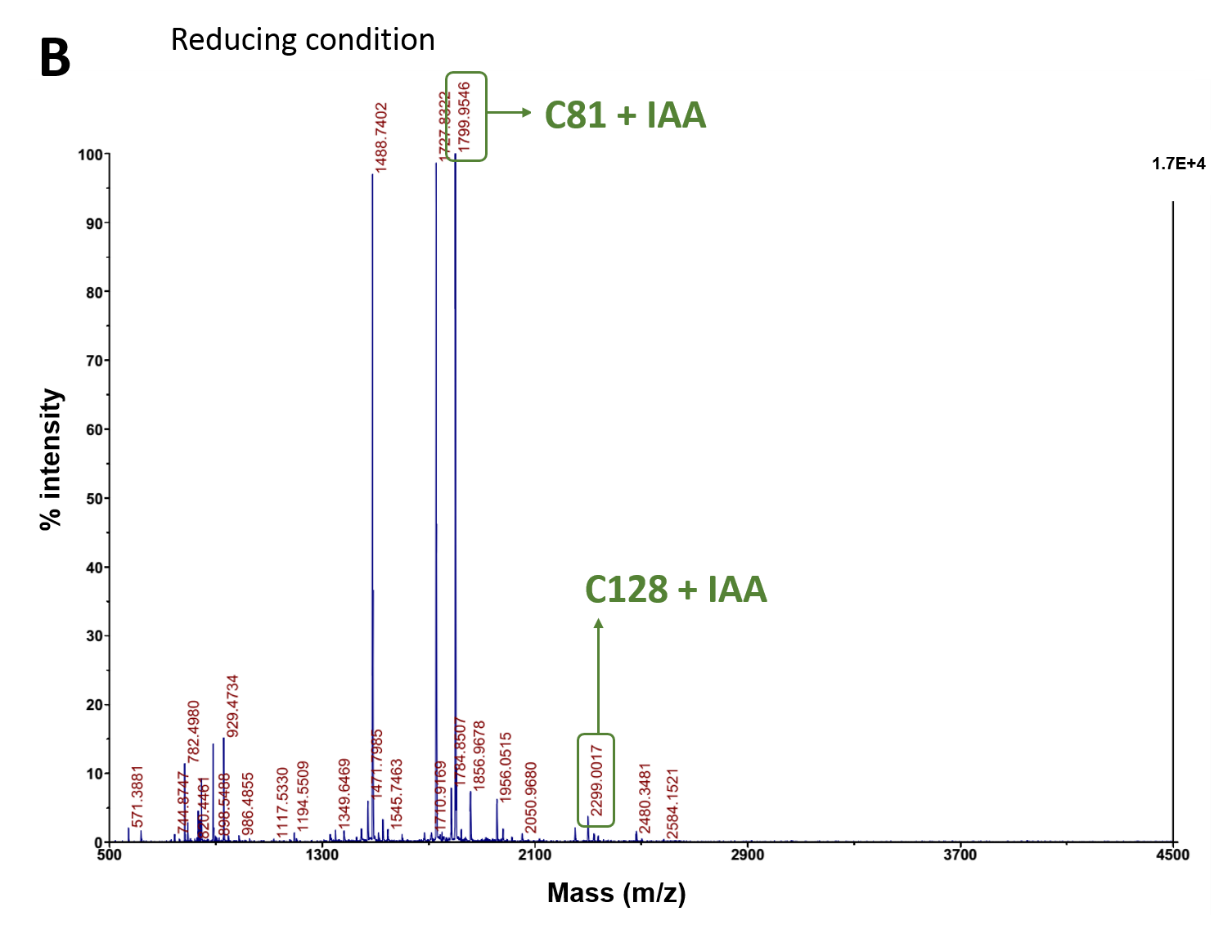


**Supplementary Figure S6.** Disulphide bond identification in His6 fusion-derived OSM (dimer fraction) under non-reducing (A) and reducing conditions (B).


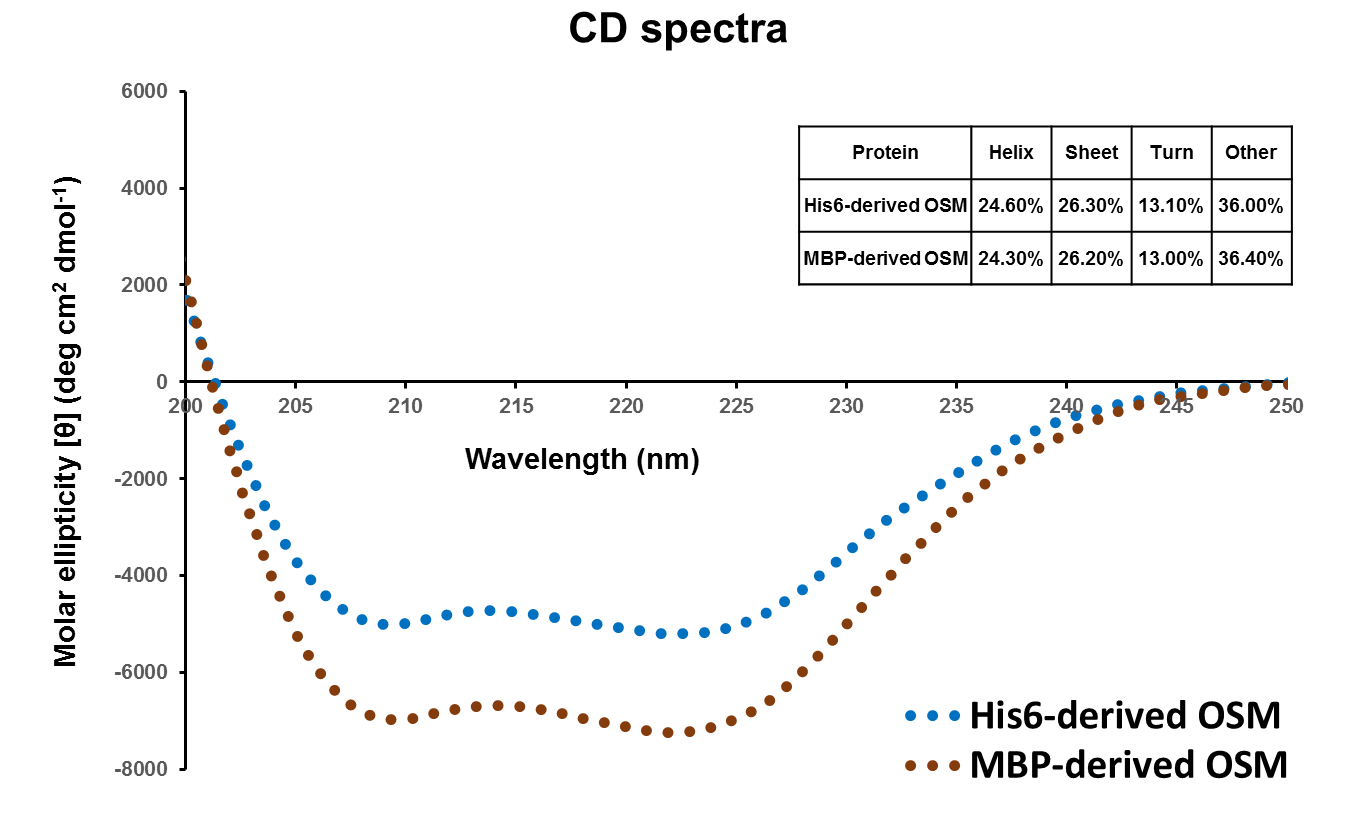


**Supplementary Figure S7.** CD spectra of the monomeric OSM proteins plotted as molar ellipticity (θ) versus the wavelength. The table represents the contribution of secondary structure types of the proteins estimated by CD Multivariate SSE program.
